# Exploiting High-Throughput Indoor Phenotyping to Characterize the Founders of a Structured *B. napus* Breeding Population

**DOI:** 10.3389/fpls.2021.780250

**Published:** 2022-01-05

**Authors:** Jana Ebersbach, Nazifa Azam Khan, Ian McQuillan, Erin E. Higgins, Kyla Horner, Venkat Bandi, Carl Gutwin, Sally Lynne Vail, Steve J. Robinson, Isobel A. P. Parkin

**Affiliations:** ^1^Agriculture and Agri-Food Canada, Saskatoon, SK, Canada; ^2^Department of Computer Science, University of Saskatchewan, Saskatoon, SK, Canada

**Keywords:** *B. napus*, spring-type, NAM, semi-automated image analysis, machine learning, drought resistance

## Abstract

Phenotyping is considered a significant bottleneck impeding fast and efficient crop improvement. Similar to many crops, *Brassica napus*, an internationally important oilseed crop, suffers from low genetic diversity, and will require exploitation of diverse genetic resources to develop locally adapted, high yielding and stress resistant cultivars. A pilot study was completed to assess the feasibility of using indoor high-throughput phenotyping (HTP), semi-automated image processing, and machine learning to capture the phenotypic diversity of agronomically important traits in a diverse *B. napus* breeding population, SKBnNAM, introduced here for the first time. The experiment comprised 50 spring-type *B. napus* lines, grown and phenotyped in six replicates under two treatment conditions (control and drought) over 38 days in a LemnaTec Scanalyzer 3D facility. Growth traits including plant height, width, projected leaf area, and estimated biovolume were extracted and derived through processing of RGB and NIR images. Anthesis was automatically and accurately scored (97% accuracy) and the number of flowers per plant and day was approximated alongside relevant canopy traits (width, angle). Further, supervised machine learning was used to predict the total number of raceme branches from flower attributes with 91% accuracy (linear regression and Huber regression algorithms) and to identify mild drought stress, a complex trait which typically has to be empirically scored (0.85 area under the receiver operating characteristic curve, random forest classifier algorithm). The study demonstrates the potential of HTP, image processing and computer vision for effective characterization of agronomic trait diversity in *B. napus*, although limitations of the platform did create significant variation that limited the utility of the data. However, the results underscore the value of machine learning for phenotyping studies, particularly for complex traits such as drought stress resistance.

## Introduction

Ensuring and increasing food security for a growing global population faced with uncertain environmental changes is one of the main challenges of agricultural research in the 21st century (Tilman et al., 2011; Hickey et al., 2019; Lenaerts et al., 2019). In addition to increasing the productivity of current arable land, it will be crucial to increase crop yield to meet rising global demand; however, yield improvement is currently progressing at an insufficient pace (Tilman et al., 2011; Ray et al., 2013). Even though technological advances, such as genomic selection and doubled haploid technology, have resulted in substantial acceleration of the breeding process, development of new high yield, pest and disease resistant, and climate-smart crop varieties is still hampered by several factors. These include long generation times and time-consuming steps such as phenotypic evaluation of large populations (Hickey et al., 2019). Accurate phenotyping, in particular, is considered one of the major bottlenecks of modern crop breeding, which has led to a strong emphasis on the development of automated, scalable, non-destructive, and high-throughput imaging approaches (High-Throughput Phenotyping, HTP). HTP could augment traditional methods of phenotypic trait quantification that are time and labor intensive, subjective, and often destructive. Over recent years, HTP applications have seen dramatic advances afforded by the continual improvement to sensor and automation technologies (Yang et al., 2020). In addition to corresponding advances in data acquisition, one of the biggest innovations in the field of HTP is the use of machine learning for automated analysis of the vast amounts of data generated by HTP platforms (Zhao et al., 2019). However, application of machine learning to the problem of phenotyping is still in its infancy and widespread deployment of these tools will require further refinements to algorithms and analysis pipelines.

Many pressing challenges with regards to crop phenotyping remain, including the measurement of multigenic or multidimensional traits and the dissection of complex phenotypes that are hard to reliably reproduce in field settings, such as abiotic stress responses, which are often the culmination of multiple environmental factors acting simultaneously (Yang et al., 2020). Some of these challenges can be overcome using indoor phenotyping systems, where growing conditions and imaging parameters can be controlled more precisely allowing individual plant architectural and physiological traits to be measured with greater accuracy. This renders controlled environment phenotyping particularly suitable for forward and reverse genetics, as well as quantitative genetics and genetic mapping (Mir et al., 2019; Yang et al., 2020). However, due to the limited space available in the small number of existing large-scale indoor phenotyping facilities as well as the high costs associated with running these experiments, only a handful of annotated benchmark image datasets are currently publicly available, mostly in Arabidopsis and grain crops (e.g., Fahlgren et al., 2015; Choudhury et al., 2016; Cruz et al., 2016; Minervini et al., 2016; Veley et al., 2017^1^,^2^).

*Brassica napus* is a multipurpose crop of major economic importance, especially the oilseed morphotype, which is a source of vegetable oil for human consumption, industrial feedstock, and protein rich meal used in animal feed (Friedt et al., 2018). Due to its relatively recent allotetraploid origin, *B. napus* has a narrow genetic base which was further eroded during initial domestication and extensive breeding activities throughout the last century (Diers and Osborn, 1994; Becker et al., 1995; Rahman, 2013; Gazave et al., 2016). Similar to other crops, this erosion needs to be addressed and further crop improvement will require the introduction of new genetic variation into current elite cultivars (Rahman, 2013; Friedt et al., 2018; Rebetzke et al., 2019). Among other approaches, this will necessitate the systematic screening of diverse germplasm collections for desirable phenotypic traits as well as extensive pre-breeding activities. In addition, dissection of the genetic basis for key traits targeted in rapeseed breeding is considered crucial for accelerated crop development (Knoch et al., 2020). Development of automated HTP protocols optimized for *B. napus*, paired with effective genomic trait dissection strategies, holds great promise to accelerate the achievement of breeding targets, such as short crop cycles, high yield, and resistance to heat and drought conditions (Delourme et al., 2018). However, comprehensive, publicly available indoor HTP datasets of *B. napus* are currently limited to 2D root phenotyping (Thomas et al., 2016a,b; Zhang et al., 2016) and early stage phenotyping (Kjaer and Ottosen, 2015; Pommerrenig et al., 2018; Knoch et al., 2020), thus the full potential of HTP technologies for above ground trait quantification has yet to be explored.

Here, we used a diverse panel of 51 spring-type *B. napus* lines, selected as founders for the development of a large, spring-type *B. napus* Nested Association Mapping (NAM) population called “SKBnNAM,” to test the feasibility of using current indoor HTP technology for rapid, semi-automated phenotyping of several key traits, including flowering and canopy architecture traits (timing of anthesis, number of flowers, and number of raceme branches) and resistance to drought. NAM is a powerful trait dissection strategy that combines association and linkage genetic mapping with genome sequencing, and it has been used to elucidate the underlying genetic architecture of several important agronomic traits in other crops, such as maize (Yu et al., 2008), barley (Maurer et al., 2015), and soybean (Song et al., 2017). The work provides a comprehensive reference image dataset for *B. napus*, as well as computational methods that can be used to extract maximal information from such datasets, including a novel method for prediction of drought stress. The work also exposes some limitations of current HTP platforms, which should be considered when considering such an approach.

## Materials and Methods

### NAM Founder Selection and Genotyping

A total of 297 *B. napus* lines from global breeding collections were genotyped using the *Brassica* 60K Illumina Infinium array as described in Clarke et al. (2016) (Supplementary Table 3.1). The genotype data was visualized using the GenomeStudio software suite (Illumina, Inc.) and a custom cluster file developed for *B. napus* was applied to screen out poorly performing and multi-locus SNPs as described in Clarke et al. (2016). The data was filtered for a minor allele frequency of 0.05 and a minimum separation of the homozygous A and B clusters of 0.8 to eliminate monomorphic and multi-locus SNPs resulting in a total of 30,933 SNPs for 297 accessions. PCA was carried out using the R package SNPRelate (Zheng et al., 2012). Vcftools v.0.1.16 (Danecek et al., 2011) was used for additional data filtering (maximum missing proportion of 0.8).

A total of 51 spring-type (or annual) lines were selected from the larger dataset to establish a *Brassica napus* NAM population (SKBnNAM) (Supplementary Table 3.2). Founder line selection was based on an assessment of levels of heterozygosity, relatedness, available phenotype data, as well as geographic origin. The chosen founder lines encompassed material from countries where spring rapeseed production is an economic priority and these were augmented with material from a diverse geography as well as the inclusion of synthetic rapeseed. The genotype data allowed the selection of the most diverse array of inbred material. The line N99-508 (NAM 0) was selected as the common parent for the NAM population. N99-508 is adapted to the Canadian environment, which potentiates the evaluation of the NAM population under Canadian field conditions.

### LemnaTec Dataset

**FIGURE 1:**
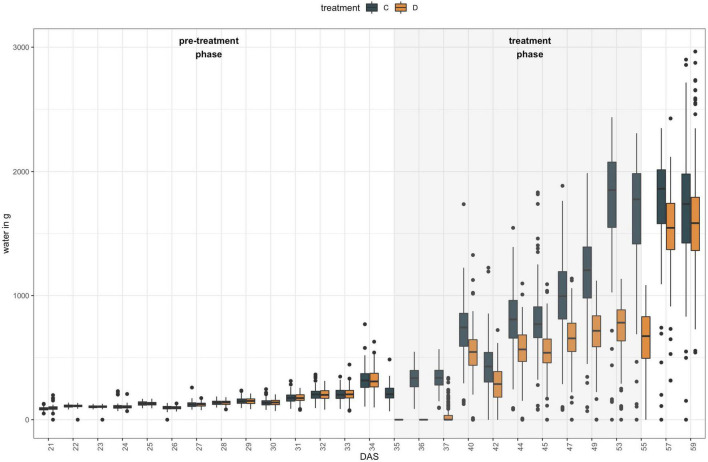
Experimental set-up of high-throughput indoor phenotyping of 50 *B. napus* founder lines at LemnaTec Scanalyzer 3D facility at University of Nebraska. Six replicates were grown of each genotype and three plants were subjected to one of two watering regimes between 35 DAS and 55 DAS: control plants (C) were kept at 100% field capacity (blue bars), while drought treated (D) plants were maintained at 40% field capacity during that period (orange bars).

Plants were grown in the LemnaTec Scanalyzer 3D facility at University of Nebraska, Lincoln campus. In total, 50 out of 51 NAM founder lines were grown in six replicates, as NAM 10 failed to germinate. All plants were sown, and grown at 18–22°C for 20 days (for detailed growing conditions, see Supplementary Table 3.3) before being loaded onto the phenotyping platform. From 21 days after seeding (DAS) to 34 DAS, plants were weighed before and after watering, and imaged once a day. During the treatment phase (35–55 DAS), three plants each were subjected to one of two different watering regimes (A: control, 100% field capacity and B: drought treatment at 40% field capacity) and imaged and weighed every day for the first 3 days, then every other day (Figure 1). During the post-treatment phase (57–67 DAS), all plants were watered equally and imaged and weighed every other day. At each phenotyping time point, images were captured for the following camera types and angles: Visible light (RGB) from 10 angles, Infrared from 6 angles, Near Infrared (NIR) from top view, fluorescent light (FLUOR) from 6 angles and hyperspectral wavelengths (HYP) from one angle. The full image dataset is openly available at https://p2irc-data-dev.usask.ca/dataset/10.1109.SciDataManager.2020.7284788 (Dataset name: P2IRC Flagship 1 Data). Camera specifications and more detailed descriptions of the phenotyping facility are available in Choudhury et al. (2016, 2018).

Dataset annotations (ground truths) were generated via manual evaluation and scoring of selected traits in both treatment groups. First, all images were assessed for presence of open flowers (0 = not flowering, 1 = flowering), with the earliest date of open flowers being counted as the date of anthesis. Then, the number of raceme branches was counted for each plant and phenotyping time point. Finally, plants were assessed for symptoms of drought stress using both top view and side view RGB images, using a binary scoring scheme in which healthy looking plants were scored as unstressed (0) and any plant exhibiting mild to severe drought stress (e.g., drooping leaves) was scored as stressed (1). Due to considerable time investment necessary to score this trait as well as the exploratory nature of this study, this ground truth was restricted to 49 DAS, which was toward the end of the experimental treatment phase but before the majority of plants had started to flower.

### Image Processing

**FIGURE 2:**
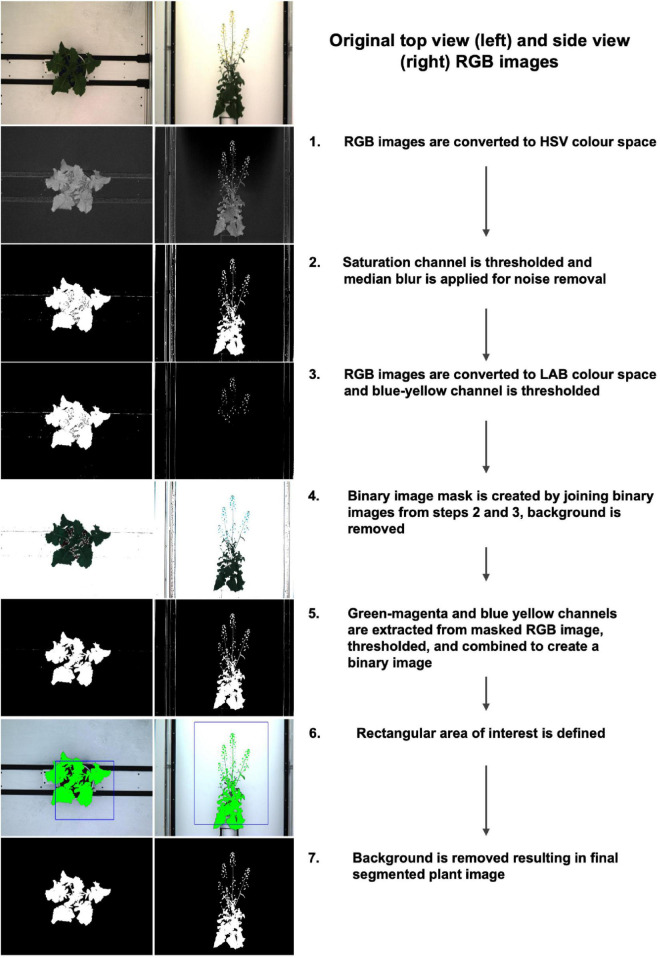
Image segmentation workflow and corresponding examples of top view (left) and side view (right) images.

For the purpose of this study, we concentrated on processing the RGB images using top view (90°) and side view (0°), and the NIR images (top view, 90°) between 21 DAS and 57 DAS. In order to remove as much background as possible, RGB images were segmented in Python 3 using PlantCV v.2 (Gehan et al., 2017) according to the steps outlined in the program documentation (Figure 2, see Supplementary Appendix 1 for extended methods). In short, this procedure involves converting the RGB image to the HSV (Hue, Saturation, and Value) and the CIELAB color space, followed by several thresholding steps, and the creation of a binary mask which is then applied to the original image to retain only plant-related information. Although manual identification of a range of thresholds made the segmentation process semi-automated, the lack of available ground truth data was the primary reason for not using automated segmentation methods with machine learning approaches. There were 300 plants and ca. 28 imaging days including both side and top view images for a total of 15988 images. Annotating these images would have been significantly more time consuming than finding the appropriate set of thresholds. Note that the segmentations were evaluated visually, by making graphs of extracted convex-hull of the plant areas. The graphs and images of 300 plants were visually checked to iteratively improve segmentation and finalize threshold values. Following image segmentation, several basic, holistic phenotypic values were extracted from each image which allowed for a numeric characterization of each plant at each phenotyping time point: the convex hull area of each plant from both top view and side view, the number of plant pixels (projected leaf area) from both top view and side view, and the plant height and width. In addition, in order to track minor leaf color changes, which could be indicative of drought stress, the Excess Green Index (ExG = 2G – R + B, Woebbecke et al., 1995) was extracted from the segmented RGB images.

**FIGURE 3:**
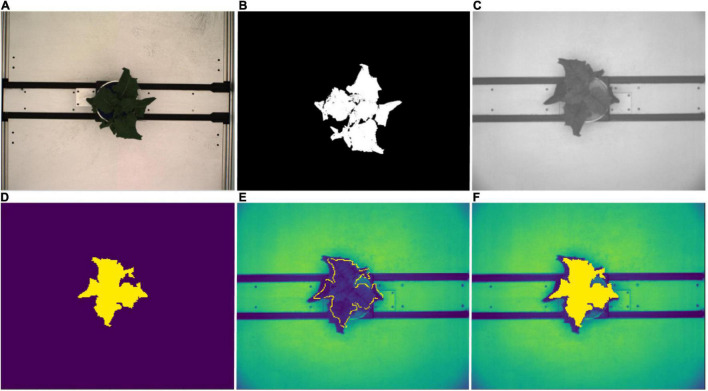
NIR image processing workflow. **(A)** Top view RGB image. **(B)** Corresponding image segmentation resulting from workflow described in Figure 2. **(C)** Original top view NIR grayscale image. **(D)** Resized and rescaled RGB image segmentation used as an image mask for NIR image. **(E)** Segmented plant outline overlaying NIR image (yellow boundary). **(F)** Segmented plant area overlaying NIR image (yellow region).

Leaf spectral reflectance is known to be substantially driven by leaf water content and NIR wavelengths have been shown to be useful in tracking plant water status (Berger et al., 2010; Briglia et al., 2019). Thus image masks from the RGB top view segmentations were fitted to the NIR images in order to extract the plant pixels of the grayscale NIR images (Figure 3). Following Vello et al. (2015) and Janni et al. (2019), NIR pixel intensities were summarized using mean and 75th percentile NIR values for each plant and day of imaging. However, due to divergent fields-of-view of the RGB and NIR cameras, this was only possible between 37 DAS and 59 DAS.

The plant boundary curvatures were extracted from the segmented top view images using the histogram of curvature over scale method (HOCS; Kumar et al., 2012). This robust, and rotation-invariant shape descriptor computes the curvature at each point on the region boundary (plant boundary) at a range of different scales, and then quantizes curvature via histograms (Van Vliet and Verbeek, 1993). Boundary curvatures on 49 DAS were computed over the normalized area integral invariant (Manay et al., 2006) at 25 different circle radii (scales) ranging from 5 to 125 (increment of 5) and summarized as histograms of curvature over scale using 5 bins per histogram at each scale.

**FIGURE 4:**
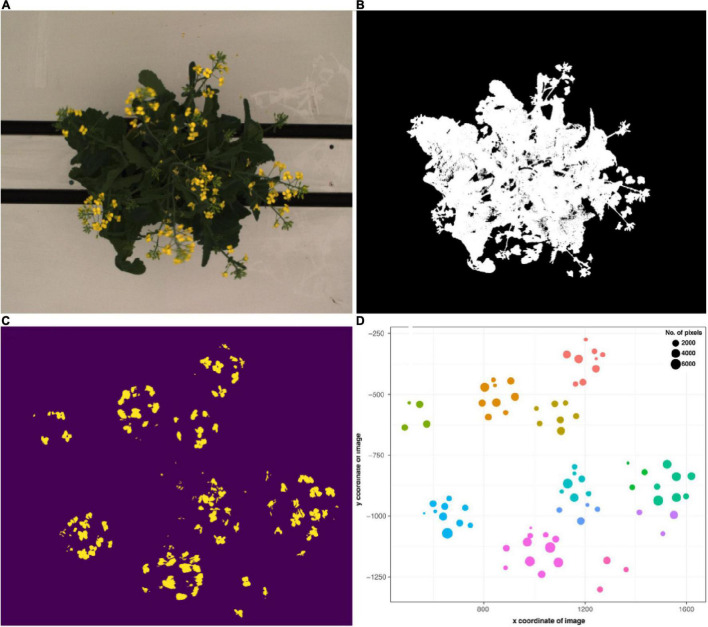
Flower and raceme branch detection workflow. **(A)** Exemplary RGB top view image of a flowering *B. napus* individual. **(B)** Segmented image. **(C)** Image components in the blue yellow channel after pixel-based thresholding. **(D)** Binary image components mapped according to their x and y coordinates and number of pixels. Colors correspond to clusters assigned via hierarchical clustering and roughly correspond to raceme branches.

Semi-automated flower detection and quantification was performed by applying the segmented RGB top view image as a mask to the blue yellow channel of the converted LAB color space top view image (Figure 4). Then, pixel-based thresholding was performed according to plant age and treatment group, and the number of pixels (“flower pixels”) in the thresholded blue yellow channel image was extracted (see Supplementary Appendix 1 for full details). The components (i.e., pixel clusters) of the resulting binary image were considered to be either single, or several overlapping flowers (Figure 4C) and their descriptive properties (number of component pixels, component centroid coordinates, minimum and maximum x and y coordinates of bounding box around each component, area of each bounding box, convex hull area) were used to plot the flowers (Figure 4D) and derive several additional properties (e.g., maximum canopy width and canopy angle). The predicted flower annotations were then compared to manual flower scores as described above.

### Data Analysis

All image processing data was analyzed under the tidyverse framework in R v. 4.0.2 (R Core Team, 2020) and RStudio v. 1.2.1335. Besides manually removing all data for plants which did not germinate, a threshold-based outlier removal procedure (median ± 3 standard deviations) was applied for each trait and day, separately (Knoch et al., 2020). In order to track day-to-day changes in plant development, day-to-day differences were calculated for most extracted traits of interest (denoted by Δ). Further, image-based phenotypes were used to derive estimated biovolume [√(side projected leaf area^2^ * top projected leaf area); Junker et al., 2015], which has previously been shown to be a suitable proxy for plant biomass in canola (Knoch et al., 2020). In addition, the results from the flower detection protocol (see above) were used to extract the maximum canopy width and the approximate canopy angle in order to identify genotypes with particularly loose or particularly compact inflorescences, a common consideration for breeders.

The number of components in the blue-yellow channel, excluding those of less than 100 pixels, was used to estimate the number of flowers per plant and imaging day. Large components, that likely included several overlapping flowers, were split by dividing their total number of pixels by the approximate number of pixels of a fully opened flower viewed from the top. Hierarchical clustering was applied using the hclust function in R using the extracted coordinates of the filtered flower components (min x, max x, min y, max y, centroid x, and centroid y) followed by pruning the resulting hierarchical tree at the total number of manually assessed raceme branches (i.e., groups, k). Spearman correlation coefficients and significance levels for selected traits were calculated in R using cor.test. Processed trait data was visualized using the R package ggplot2 (Wickham, 2016).

### Machine Learning

Several supervised machine learning models were tested for prediction and identification of two key agronomical traits: the number of raceme branches and drought stress. The manually assessed ground truth of the number of raceme branches consisted of 789 observations from 39 genotypes that started flowering within the experimental period with 34 different class labels (number of raceme branches). Since raceme branch counts were unevenly distributed [e.g., 198 of 789 images were of flowering individuals with one inflorescence branch (main stem) while only 21 images featured plants with 10 raceme branches], this was considered a regression rather than a classification problem. The data set was randomly divided into 5 sets for a 5-fold cross validation, with 4 sets serving as training data and one set serving as testing data in 5 replicate experiments. For each set, plant age in DAS, the filtered number of pixels in the blue-yellow channel (i.e., “flower pixels”) and the estimated number of flowers were used as input features for raceme branch number prediction. The widely used linear regression and Huber regression algorithms were applied to predict the inflorescence branch numbers (Huber, 1992; Freedman, 2009). Prediction accuracy of different machine learning algorithms was then gauged by comparing to the manually established ground truth of raceme branch numbers.

With regard to drought stress, the number of stressed plants (*n* = 166) was randomly down-sampled to match the number of unstressed plants (*n* = 133), resulting in a total dataset of 266 observations. Combinations of several input features were used to test machine learning for stress detection, including mean and 75th percentile NIR, difference in plant pixels between 47 DAS and 49 DAS (Δ pixelsTV, assuming that drooping leaves might result in a reduction in plant size as seen from above), the convex hull area (top view), total number of plant pixels (top view), Δ convex hull (top view), plant height and width, Δ plant height and Δ plant width, ExG, and number of raceme branches. Again, the data set was randomly divided into 5 subsets for a 5-fold cross validation, with 4 sets serving as training data and one set serving as testing data in 5 replicate experiments. Stress identification was tested with the commonly used supervised machine learning algorithms Random Forest Classifier, Linear Discriminant Analysis, Logistic Regression model, K-nearest Neighbor, Decision Tree, and Support Vector Machine, most of which had been previously employed for stress detection in plants (Singh et al., 2016).

## Results

### The *Brassica napus* Germplasm

**FIGURE 5:**
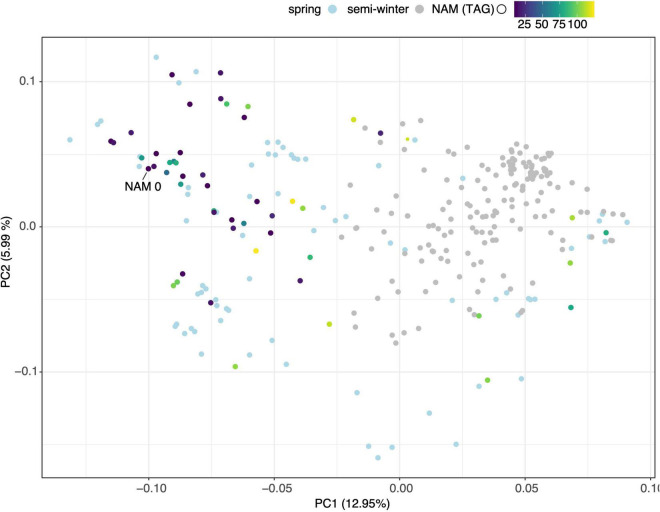
Principal Component Analysis of genotypic diversity of 297 spring (light blue dots) and semi-winter (gray dots) *B. napus* varieties. Founder lines of SKBnNAM introduced in this study are colored according to their mean total aliphatic glucosinolate (TAG) levels.

**FIGURE 6:**
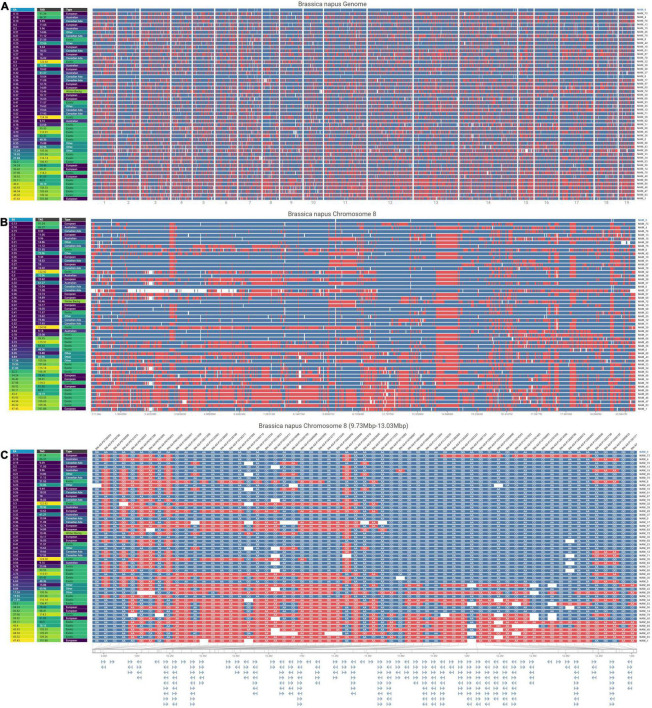
Visualizations at three resolutions comparing SNPs in 50 *B. napus* genotypes against a reference genotype (NAM 0) with the genotypes ordered by their increasing erucic acid content. The visualizations are all organized with rows representing different lines, and columns of pixels representing each SNP. The color at each SNP indicates whether that SNP is the same (blue) as or different (red) from the allele in the reference genotype shown at the top of the view and missing data is encoded in white. Additionally, at each resolution, a map of phenotypic trait values (erucic acid content, total aliphatic glucosinolate levels, and genotype origin) are shown on the left. **(A)** Genome-level overview of the entire *B. napus* genome horizontally separated by chromosome. **(B)** Chromosome-level view of chromosome 8 in *B. napus*. **(C)** SNP-level view of chromosome 8 (9.73 mb to 13.03 mb) that displays SNP names at the top of the column, nucleotides for each SNP, and a gene map below the main view.

A total of 30,933 single nucleotide polymorphism (SNP) markers assayed from the Illumina *Brassica* Infinium SNP array were used to evaluate the genetic diversity among a wide collection of *Brassica napus* lines (Supplementary Table 3.1). The first two principal components of a principal component analysis (PCA) of 297 spring and semi-winter lines jointly explained 18.94% of the genetic variance and, similar to other studies, showed that these are positioned along a genetic gradient rather than constituting discriminate clusters (Lu et al., 2019). The founder lines of the SKBnNAM were selected to be well distributed among the genotyped spring-type accessions to capture most of the available variation (Figure 5). The available pedigrees and a summary of key seed quality traits (aliphatic glucosinolate and erucic acid content) segregating among the founders are provided in Supplementary Table 3.2. The SNP data for the founder lines has been represented in a novel graphical visualization tool^3^ that allows relationships between the lines to be interrogated at the genome, chromosome and SNP level (Figure 6). The lines can be arranged according to user-specified trait data in order to facilitate exploration of underlying genetic variation.

### Plant Image Capture, Segmentation, and Trait Extraction

The initial HTP experiment intended to capture variation of the founder lines of the SKBnNAM in response to low water availability. Six replicates of each line were grown and imaged in a LemnaTec Scanalyzer 3D plant phenotyping facility (for an overview of growing conditions, see Supplementary Table 3.3). Thirty-four days after seeding (DAS) three of the replicates were maintained at field capacity (control group), while three were subjected to reduced watering to approximate 40% field capacity (treatment group). Imaging was carried out every day initially and every second day after DAS 37, and RGB images (top view, 90° and side view, 0°) as well as near-infrared (NIR) images were processed for specific phases of the experiment (see Materials and Methods). There was high variation for all extracted plant traits within genotypes and treatment groups (Supplementary Figure 3.1) and plants of both treatment groups displayed symptoms of drought stress (Supplementary Figures 3.2,3.3). This was likely caused by uneven watering administered during plant growth, mainly resulting from the large basal leaves blocking the automated watering system. Due to this variation, data analyses mainly focused on assessing and improving the efficacy of semi-automated image analysis protocols. Whenever genotype-specific traits were extracted (e.g., flowering time and canopy traits), analysis was limited to the control group.

**FIGURE 7:**
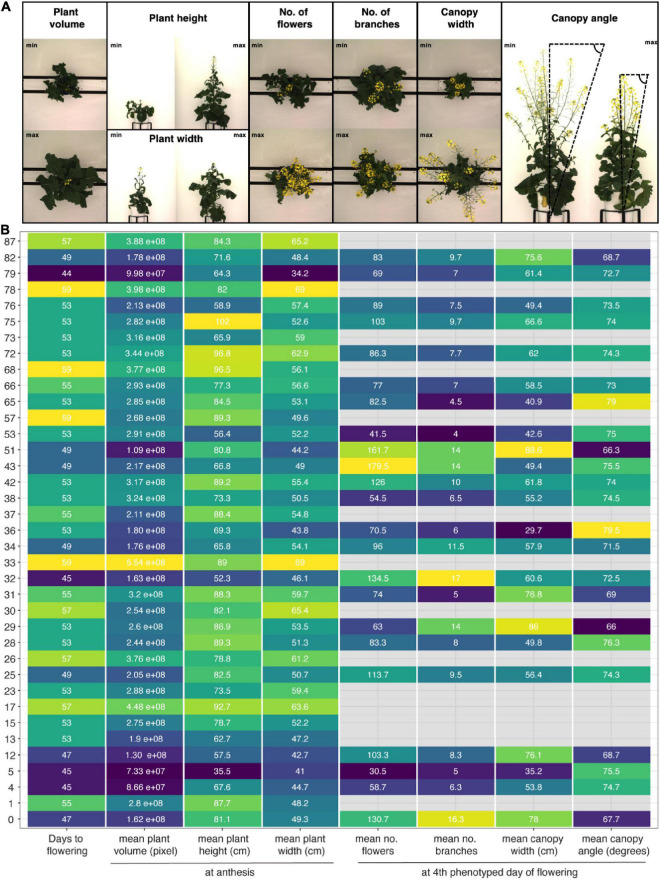
Morphological diversity among founder lines of SKBnNAM based on eight selected growth traits extracted from the dataset. **(A)** Images show individuals representing minimum and maximum values for each growth trait (except for days to flowering). **(B)** Mean values for each trait and genotype (control group only). Tiles are colored based on normalized trait means (darker colors indicating lower values, lighter colors indicating higher values). Absolute mean values are given in each tile. Only genotypes that started to flower before DAS 59 are included in this plot (*n* = 37).

Despite these challenges, the described image analysis pipeline allowed for extraction of holistic and derived phenotypic traits, including plant height, plant width and plant volume, a proxy for biomass, as well as plant phenology and canopy architecture traits (Figure 7 and Supplementary Figure 3.4A, Supplementary Appendix 2). The method efficiently captured the wide-ranging diversity of growth and flowering traits among 50 out of 51 SKBnNAM founder lines (Figure 7), thereby demonstrating the potential of the population. While the exact thresholds used in the image segmentation and trait extraction process (Supplementary Appendix 1) are specific to this particular experiment, the outlined process used to derive these thresholds can be applied for other crops and experimental setups.

### Flower Detection and Quantification

**FIGURE 8:**
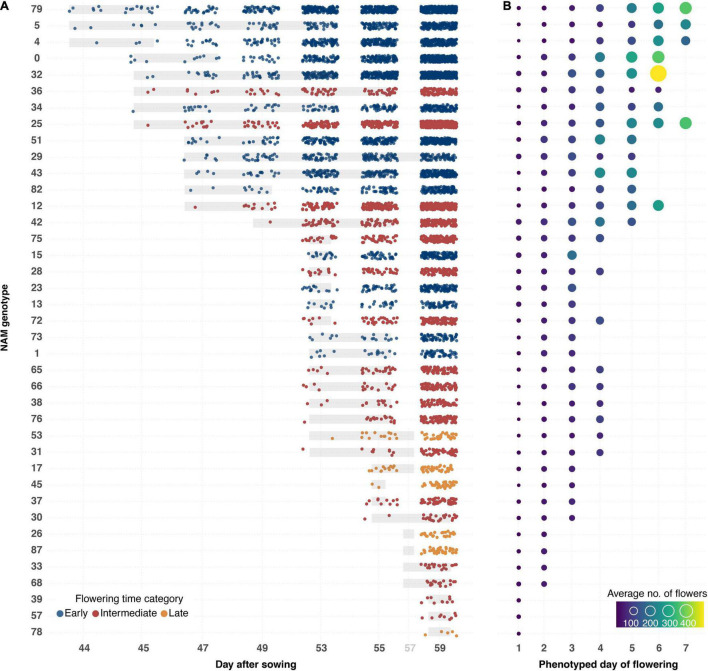
Flowering phenology and intensity for 50 *B. napus* genotypes ranked by date of anthesis and flower output. **(A)** Flowering onset and intensity of *B. napus* genotypes that flowered during the LemnaTec experiment. Dots represent average estimated number of flowers recorded from three individuals (control group) of each genotype. The dots are colored according to previous greenhouse observations of flowering time. Underlying gray bars represent variation in flowering onset. **(B)** Flowering intensity per phenotyped day of flowering. Sizes and colors of circles represent average number of flowers recorded from three individuals (control group) of each genotype. Order of genotypes is shown in left-hand column in **(A)**.

A semi-automated pipeline for flower detection was designed that allowed flowering to be accurately and precisely tracked throughout the experiment (Figures 4, 8). This pipeline involved using the segmented top view RGB image as an image mask on the blue-yellow channel image of the same view, obtained by converting the RGB image to the LAB color space. Results of automatic flower detection were compared to manually determined dates of anthesis for each plant. After identification of the appropriate thresholds (see Supplementary Appendix 1), anthesis was correctly detected in 170 out of 175 flowering plants (97.14% accuracy). In four of the five cases where detection was not accurate, flower buds were observed in which the yellow petals were protruding from the sepals (Supplementary Figure 3.5A). The last case was caused by a single flower petal that had fallen from another plant (Supplementary Figure 3.5C). Following the successful detection of anthesis, the number of pixels as well as the number of components in the segmented image of the blue yellow channel (Figure 4C) were used to estimate the number of flowers for each plant and phenotyping time point. This approach allowed the flowering period of each plant to be measured through time, and for the different genotypes to be compared in terms of their phenology, flowering intensity, and variability among these characteristics (Figure 8). Early flowering varieties were clearly identified and the flowering behavior of these genotypes was consistent with those measured from previous field characterization (Spearman’s rho = 0.76, Supplementary Table 3.2).

In order to facilitate the description of canopy architecture, quantification of raceme branches was explored by using the output from the flower detection pipeline as input to supervised machine learning algorithms; the results were then compared against manually counted raceme branch numbers for every plant at each phenotyped flowering time point. It was found that raceme branch numbers could be predicted from the estimated number of flowers and the total number of flower pixels with an accuracy of 91% ± 0.02 with both tested machine learning algorithms (Linear regression and Huber regression, both with 5-fold cross validation, Table 1). As some input features were correlated [flower pixels and number of flower components (estimated number of flowers), Spearman’s correlation coefficient: r^2^ = 0.99], the prediction was tested without the flower pixels feature and achieved 90% ± 0.03 accuracy. Overall, the number of the flower components feature seemed to have more impact on branch prediction accuracy than the flower pixels feature. Consecutively, individual components from the thresholded blue-yellow channel were assigned to individual raceme branches (Figure 4D), by using hierarchical clustering. Taken together with additional image-derived traits such as canopy width and plant height, this allowed canopy architecture for the different genotypes included in this experiment to be broadly characterized (Supplementary Figure 3.4).

**TABLE 1:** Mean accuracies for inflorescence branch number prediction from image-derived traits for 50 *B. napus* genotypes using different machine learning algorithms.

	Raceme branch number prediction		

Number of observations (total/training data/testing data)	789/631/158		

**Mean accuracy with 5-fold cross validation (SD)**			

	**All features**	**All features except for “flower pixels”**	**All features except for estimated number of flowers**
Linear Regression	91% (±0.03)	90% (±0.04)	85% (±0.05)
Huber Regression	91% (±0.02)	90% (±0.02)	85% (±0.06)

*Total number of observations, number of observations in training data, and number of observations in testing data are given. 5-fold cross validation was used. Input features for inflorescence branch number prediction were plant age, “flower pixels,” and number of flower components (estimated number of flowers). SD = Standard deviation across cross validation replicates.*

### Drought Phenotyping

It was anticipated that withholding water from a subset of actively growing plants would result in a reduced growth rate, an increase in temperature resulting from reduced evapotranspiration, and wilting from reduced turgor pressure. In order to test semi-automated detection of these symptoms, drought stress was visually scored for all plants of both control and drought-treatment groups using a binary scheme (0: unstressed, 1: symptoms of drought stress) and the top and side view RGB images on DAS 49.

First, three individual image-based traits were tested for their efficacy as proxy traits in semi-automated drought stress detection: NIR intensity, Excess Green Index (ExG), and Δ pixelsTV (the change in the number of plant pixels from top view between DAS 47 and DAS 49, assuming that wilting would result in a smaller number of plant pixels captured from the top). However, taken on their own, none of these three traits showed substantial differences in plants displaying visible drought stress compared to unstressed plants on DAS 49 (Supplementary Figure 3.6). Similarly, two-way combinations of any of the three traits could not reliably distinguish between stressed and non-stressed plants (data not shown).

**FIGURE 9:**
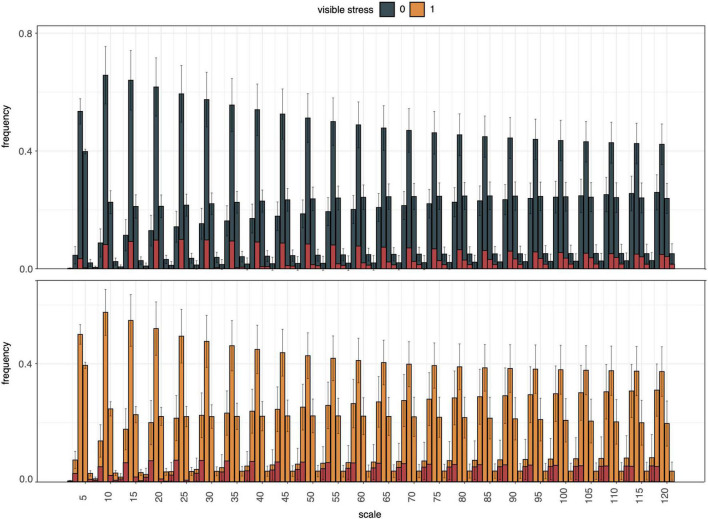
Summary of histogram of curvature over scale (HOCS) method for all unstressed (0, blue) and all visibly stressed (1, orange) individuals on DAS 49. Each plot shows a series of 25 histograms that are composed of 5 bins each summarizing the curvature of the segmentation boundary at a given scale. Error bars give standard deviations across all individuals in each group (unstressed, visibly stressed). Overall histogram distributions were visibly different between these two groups (highlighted by red column portions signifying absolute mean differences for each bin). However, high within-group variation across the diversity panel masked these between-group differences and prevented accurate drought stress prediction from curvature of segmentation boundaries alone.

Next, curvature of the segmentation boundary was tested as an indicator of drought stress. The histogram of curvature over scale (HOCS; Kumar et al., 2012) method was applied to the top view plant boundary on DAS 49, assuming that plants experiencing water deficit would exhibit rolling of leaf edges. Although the histograms of curvature of stressed and unstressed plants did exhibit differences in their proportions at different scales, their within-group variation was too high to reliably distinguish between these two phenotypes (Figure 9).

Finally, the potential of machine learning algorithms in semi-automated drought stress detection was assessed by comparing the results of supervised machine learning to the manually determined stress scores of DAS 49. Using only the curvature of the top view plant boundary (summarized by HOCS), the highest accuracy achieved in initial tests was 66.6% using the KNN algorithm (K = 1), which was judged too low to be investigated any further. In contrast, using a combination of 21 phenotypic traits and image attributes extracted from the image dataset (mean and 75th percentile NIR, total number of plant pixelsTV, Δ pixelsTV, the convex hull areaTV, Δ convex hullTV, total number of plant pixelsSV, Δ pixelsSV, the convex hull areaSV, Δ convex hullSV, weight before, and after watering, plant area below pot rim, plant height, plant width, Δ plant height (between DAS 47 and 49), Δ plant width, ExG, flowering group (early, intermediate, late), and number of raceme branches) with several supervised machine learning algorithms was able to detect drought stress with the area under the receiver operating characteristic curve (ROC AUC) > 0.8 with 5-fold cross validation (Table 2). The maximum mean ROC AUC was achieved using the Random Forest algorithm (0.85 ± 0.03), Linear Discriminant Analysis (0.82 ± 0.04), and Logistic Regression (0.82 ± 0.08) algorithms. We also calculated the accuracy and achieved a maximum mean accuracy of 81% for 5-fold cross validation with the Random Forest Algorithm. Removing single features resulted only in very small changes to overall identification of ROC AUC in most cases (Supplementary Table 3.4).

**TABLE 2:** Mean ROC AUC, and mean accuracies for drought stress identification from image-derived traits for 50 *B. napus* genotypes using different machine learning algorithms.

	Drought stress identification	

Number of down-sampled observations (total/training data/testing data)	266/212/54	

	**Mean ROC AUC with 5-fold cross validation**	**Mean accuracy with 5-fold cross validation (SD)**
Random Forest	**0.85**	**0.81 (±0.03)**
Linear Discriminant Analysis	0.82	0.77 (±0.04)
Logistic Regression	0.82	0.79 (±0.08)
Decision Tree	0.73	0.75 (±0.12)
KNN classifier (K = 1)	0.72	0.73 (±0.08)
Support Vector Machine (SVM)	0.76	0.67 (±0.23)

*Total number of observations, number of observations in training data, and number of observations in testing data are given. 5-fold cross validation was used. Input features for drought stress identification on 49 DAS were mean and 75th percentile NIR, total number of plant pixelsTV, Δ pixelsTV, the convex hull areaTV, Δ convex hullTV, total number of plant pixelsSV, Δ pixelsSV, the convex hull areaSV, Δ convex hullSV, weight before, and after watering, plant area below pot rim, plant height, plant width, Δ plant height, Δ plant width, ExG, flowering group (early, intermediate, late), and number of raceme branches. All Δ plant values represented differences between 47 DAS and 49 DAS. ROC AUC = Area Under the Receiver Operating Characteristic Curve. SD = Standard deviation across cross validation replicates. Achieved maximum results have been highlighted in bold.*

## Discussion

The potential of HTP combined with automated image processing and machine learning for accelerating crop improvement has been increasingly acknowledged; however, deployment is currently hampered by the lack of reliable, robust, and easy to implement data processing methods. The results presented here show that scoring of basic plant traits, such as plant height and width, but also more complex traits such as flowering traits and canopy architecture in a streamlined, semi-automated fashion can be reliably achieved, once a workflow has been optimized. In addition, the potential of machine learning to identify multi-dimensional plant phenotypes such as drought stress resistance was shown. This methodology thus shows great promise for application in targeted breeding programs for *B. napus* and other crops.

### Introduction of the Diverse Founder Panel for SKBnNAM

Since their introduction more than a decade ago, NAM populations have proven to be invaluable tools for the dissection of the genetic architecture of complex traits and have been adopted for a number of economically important crops (Yu et al., 2008; Gage et al., 2020). The lines phenotyped in this study form the founder panel for a new spring-type *B. napus* NAM population, SKBnNAM. Besides the common founder, N99-508, this panel includes seven Canadian adapted, five Australian, 13 European, 18 Asian as well as seven other (e.g., Argentinian and yellow-seeded) lines (Supplementary Table 3.2), which capture the genetic spectrum of spring-type *B. napus* (Figures 5, 6). This population represents a useful complement to the previously published Bn-NAM population (16 founder lines, 2425 F_6_ recombinant inbred lines) which was developed from mostly semi-winter-type rapeseed, a morphotype that is predominantly grown in China (Hu et al., 2018), as well as to a large *B. napus* NAM population developed from winter and synthetic lines (51 founder lines, Snowden et al., 2015). Based on preliminary genotype data and available field phenotype data it was expected that SKBnNAM would be a valuable resource for dissecting the genetic architecture of complex traits in *B. napus*, and thus be instrumental for rapeseed crop improvement. However, it was still necessary to establish a clear picture of the level of phenotypic variation potentially segregating within the population.

### Considerations for Controlled Environment HTP Experiments in *B. napus*

The data presented represents one of the longest controlled environment HTP datasets for *B. napus* published to date. While this dataset will certainly become a valuable resource for image analysis refinements and innovations in rapeseed phenotyping, experimental problems that were encountered should be taken into consideration for future experiments. As mentioned above, higher than expected within genotype × treatment variation suggested that watering regimes were not administered as precisely as planned, leading, for example, to some individuals of the control group exhibiting symptoms of drought stress (Supplementary Figures 3.2,3.3). This was likely due to a combination of relatively small pot sizes (9 L pot volume, Supplementary Table 3.3) and automated overhead irrigation which led to larger rosette leaves effectively shielding the soil in some individuals. In addition, the automated plant-to-sensor movement within the phenotyping facility presented additional challenges during the experiment, as some plants were damaged (some larger, overhanging rosette leaves were torn, Supplementary Figure 3.3) while being moved through the system. Most of these challenges are connected to the plant architecture of *B. napus* as compared to other crop species frequently used in indoor HTP experiments (e.g., maize and rice) which suggests that scaling this system to different crop species requires crop-specific expertise and adjustments. Despite these challenges it was possible to focus on plant traits that were unaffected and further test the feasibility of different image analysis approaches for *B. napus*.

### Flower Phenotyping/Growth Stages

Major breeding objectives in *B. napus* include early germination, improved seedling root development, early onset of flowering, high yield, favorable aerial plant architecture, reduced pod shatter, improved seed oil content, and quality as well as tolerance to biotic and abiotic stresses (Delourme et al., 2018). A focus of the current study was the semi-automated evaluation of flowering traits, plant aerial architecture and, where feasible, resistance to drought stress. It was demonstrated that it is possible to efficiently and accurately track flowering time and intensity through time using overhead RGB imaging (Figure 8). While we are not aware of any previous indoor HTP studies of *B. napus* that proposed flower detection and quantification to a similar degree of precision as this one, the approach presented here was similar to the one outlined by Chen et al. (2019) who separated flower volume from plant volume on the basis of pixel color. However, flowering traits were not the primary focus of their study and the authors did not investigate the possibility of using this method to track flower timing throughout their experiment. The results demonstrated that anthesis in *B. napus* does not need to be scored manually in greenhouse settings [as done by Chen et al. (2019)] but can be accurately detected semi-automatically (97.14% accuracy). This represents a significant extension of previous approaches as it further allows us to approximate flower numbers per plant and phenotyping time point. Accurate flower tracking also enabled the characterization of plant aerial architecture throughout the growing period (e.g., canopy width, canopy height, canopy angle, as well as number of raceme branches, Figure 7, and Table 1). Although not possible in the current experiment, the same methodology could easily be applied to capture the end of the flowering period and thus compare the length of the flowering period across genotypes and treatments. Semi-automated and eventually fully automated detection of flowering will facilitate high-throughput germplasm screening for early flowering varieties as well as extraction of other informative image traits or spectral indices that are scored during flowering, for example leaf spectral features which have been shown to be strongly correlated to seed yield (R^2^ = 0.71) when measured during the flowering stage (Zhang and He, 2013). Due to a number of additional factors (e.g., in-field variability of growing conditions, presence of parasites/disease agents, spectral signal of canopies rather than single plants, and shadows from overlapping plants), field phenotyping requires a slightly different approach to automated tracking of flowering in rapeseed. However, similar to our findings, the blue and green spectral bands of RGB images (e.g., used to calculate the normalized difference yellowness index, NDYI) have proven to be the most useful for distinguishing between flower and overall canola canopy signals in field settings (Sulik and Long, 2020).

### Drought Phenotyping

Drought stress caused by moderate loss of water is characterized by a reduction in water content, diminished leaf water potential and turgor loss, accompanied by stomata closure, and decrease in cell enlargement and growth, while desiccation (severe water stress) can result in arrest of photosynthesis and even plant death (Jaleel et al., 2009). However, there is considerable plasticity in the degree to which water loss can be minimized and high-water status can be sustained among and within species (Farooq et al., 2013). This can lead to a broad spectrum of drought stress symptoms among closely related crop varieties. These wide-ranging symptoms are often visually scored by experienced breeders, but this integrated task is much harder to translate into automated stress detection protocols.

**FIGURE 10:**
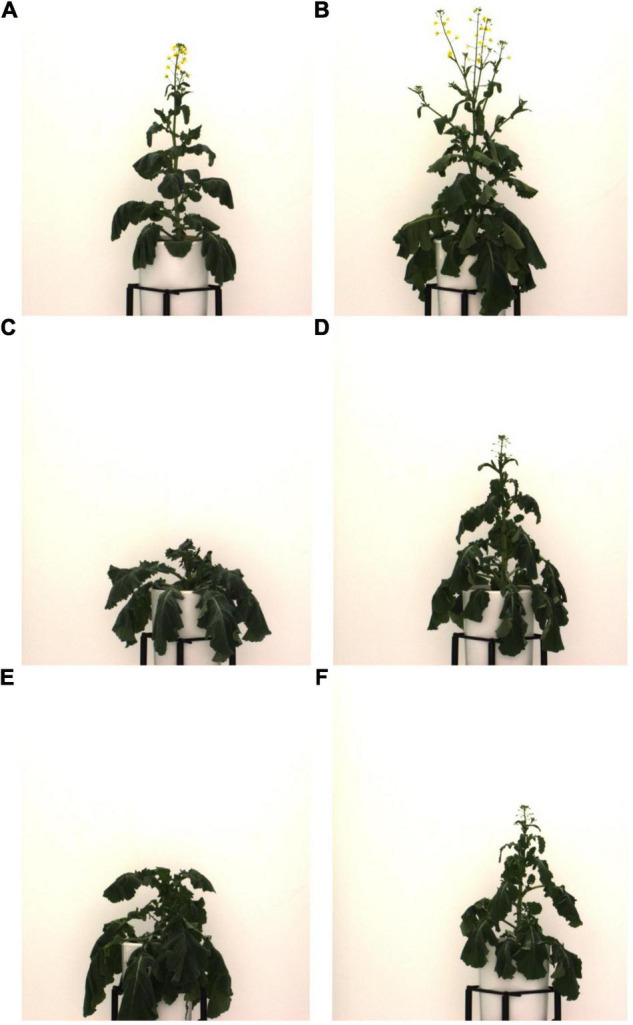
Examples of *B. napus* individuals exhibiting mild to moderate drought stress on 47 DAS. All images show plants from control group. For further examples of drought stress symptoms, see Supplementary Figure 3.3. **(A)** NAM 12, Ind 423; **(B)** NAM 0, Ind 497; **(C)** NAM 30, Ind 492; **(D)** NAM 75, Ind 422; **(E)** NAM 33, Ind 479; and **(F)** NAM 75, Ind 457.

Using single or dual image-based plant traits such as NIR intensities, ExG (a measure of greenness), and daily changes in projected leaf area (Δ pixelsTV) were not reliable ways to identify drought stress in our experiment (Figure 6 and Supplementary Figure 3.6). This is in contrast to findings from other indoor phenotyping experiments. For example, Janni et al. (2019) found significantly different NIR intensities and greenness values for drought-stressed and well-watered tomato plants. Similarly, Vello et al. (2015) found that they could accurately identify *A. thaliana* individuals in a water-limited treatment group using an NIR intensity threshold. Finally, Briglia et al. (2019) reported a clear correlation between stem water potential and both, NIR intensities and greenness parameters for drought-treated grapevines. One likely explanation for this difference is the degree to which drought was imposed during these experiments. While plants were often stressed irreversibly, to the extent of wilting, leaf yellowing or browning (senescence), and even lethal dehydration in previous studies (e.g., Vello et al., 2015; Duan et al., 2018; Briglia et al., 2019), individuals exhibited only comparatively mild drought symptoms in our experiment (e.g., leaf rolling, loss of turgor, Figure 10, and Supplementary Figure 3.3). It thus appears that both NIR intensities and greenness indices such as ExG are more suitable for detecting severe levels of drought stress. In addition, the second half of the treatment phase coincided with the onset of flowering leading to temporal overlap in flowering and more severe drought stress. This created substantial problems for the utility of NIR for drought detection since yellow *B. napus* flowers refract NIR the same way that senescent leaves would and thus, flowering-dependent changes in NIR intensities effectively mask the effects of drought stress (also seen by a significant positive relationship between NIR intensity and the number of flower pixels, Spearman’s rank correlation rho = 0.38, Supplementary Figure 3.7A). Unsurprisingly, ExG was also driven by presence of flowers (rho = −0.68, Supplementary Figure 3.7B). Thus, the suitability of previously applied image-based traits for measuring drought stress strongly depends on experimental timing of drought stress and flowering in *B. napus*. Finally, very few previous drought phenotyping studies included diversity panels of the studied crops. The genotypic variation in leaf color, leaf thickness, leaf shape and size, and plant size made it impossible to define common thresholds of the tested parameters that would have allowed accurate semi-automated drought stress identification across all 50 assayed *B. napus* lines (Supplementary Figure 3.8). This illustrates the limitations of using these traits in complex experimental settings, such as diverse pre-breeding trials.

Instead, the data revealed the promise of supervised machine learning for stress identification in such complex situations. To the best of our knowledge, plant boundary curvature as summarized by the HOCS method, was used here for the first time to evaluate drought stress in an indoor HTP experiment. While promising overall, using this metric on its own was judged to be not effective for reliably distinguishing between stressed and unstressed plants, again likely due to genotype-specific variation overriding any shared symptoms of drought stress. However, using combinations of plant traits and image attributes derived from RGB and NIR images, it was possible to achieve stress identification accuracies of more than 80%. This mirrors previous studies that reported promising results using a variety of machine learning algorithms for drought detection and prediction in other crops (e.g., Römer et al., 2012; Raza et al., 2014); however, here for the first time with the added complexity of a large diversity panel. This added complexity clearly identified drought stress to be a multi-dimensional phenotype that could not be described by single attributes or traits but instead required a combination thereof. Compared to hyperspectral imaging, which has successfully been used in drought stress identification (e.g., Römer et al., 2012; Susič et al., 2018; Asaari et al., 2019), but which can be substantially more costly and difficult to acquire, process, and store (Holzapfel, 2007), our results underline the utility of NIR and multispectral RGB for this kind of problem.

Despite these promising results, several drawbacks remain to be solved. Image data preprocessing, one of the most crucial steps for successful use of machine learning methods (Singh et al., 2016), is time-consuming and requires deep-rooted knowledge of the organism of interest for input feature selection. For example, even though several different combinations of input features were tested (Supplementary Table 3.4), it was not possible to achieve stress identification ROC AUC of >85% in this experiment. Yet, further refinement of the input feature combinations for reliable drought stress identification, for example using additional plant attributes or traits could lead to higher identification accuracies. In addition, further improvements could likely be achieved through expansion of the manually scored stress ground truth to multiple days in order to allow for better genotypic-specific trait learning. However, this would require a significant time investment as manually scoring mild drought symptoms from images can be relatively tedious and somewhat subjective even to the experienced eye. The ultimate goal of stress phenotyping is stress prediction before the onset of stress symptoms that can be distinguished by the human eye. Even though our study makes a valuable contribution to this overall problem, future work will be needed to solve the puzzle of drought stress prediction in *B. napus* and other crops.

## Conclusion

Phenotyping is considered a major bottleneck slowing the development of new crop varieties. This study illustrates the utility of semi-automated image processing and supervised machine learning for pre-breeding activities in *B. napus* by demonstrating their efficacy in scoring key agronomic traits including flowering characteristics (e.g., timing and volume), canopy architecture traits (e.g., raceme branch numbers), and early symptoms of drought stress in a diverse panel of spring lines. Despite several methodological challenges connected to scaling an indoor HTP platform to different crops, the results presented underline the promise of state-of-the-art HTP technologies, semi-automated image processing, and supervised machine learning for crop improvement, in particular when combined with genomics and systematic breeding strategies.

## Data Availability Statement

The datasets presented in this study can be found in online repositories. The names of the repository/repositories and accession number(s) can be found below: https://p2irc-data-dev.usask.ca/dataset/10.1109.SciDataManager.2020.7284788, N/A; https://genomevis.usask.ca/haplotype-map-tree/, N/A.

## Author Contributions

IP and SR conceived and supervised the original experiments and research plans. NK supervised by IM designed and performed image processing, trait extraction, and machine learning experiments. JE carried out data analysis, generated all data annotations, and wrote article with contributions from all the authors. VB and CG conceived and built the SNP data visualization tool. SV was involved in NAM founder line selection and original phenotyping. EH contributed SNP genotyping and genomic mapping data and provided technical assistance for their analysis. KH assisted with founder line selection and further population development. IP agreed to serve as the author responsible for communication. All authors contributed to the article and approved the submitted version.

## Conflict of Interest

The authors declare that the research was conducted in the absence of any commercial or financial relationships that could be construed as a potential conflict of interest.

## Publisher’s Note

All claims expressed in this article are solely those of the authors and do not necessarily represent those of their affiliated organizations, or those of the publisher, the editors and the reviewers. Any product that may be evaluated in this article, or claim that may be made by its manufacturer, is not guaranteed or endorsed by the publisher.

## Footnotes

1
https://plantvision.unl.edu/dataset

2
https://www.quantitative-plant.org/dataset

3
https://genomevis.usask.ca/haplotype-map-tree
