# Supplementary material for: Exploiting High-Throughput Indoor Phenotyping to Characterize the Founders of a Structured B. napus Breeding Population
Source: Front Plant Sci. 2022 Jan 5;12:780250. doi: 10.3389/fpls.2021.780250 (PMC8767643; doi:10.3389/fpls.2021.780250)
Supplement: Supplementary file 1 [file Data_Sheet_1.docx]

**Appendix S1: Extended Methods**

1. **Data pre-processing and image segmentation**

In a time-sequence phenotyping study, especially one focussing on stress and flower phenotyping in a diverse set of crop lines such as this one, image processing must be designed to not only capture mostly green leaves, but also differently coloured parts of the plants (e.g. reddish/brownish leaves, necrotic tissue, flowers). Here, this is accomplished by converting original RGB images to different colour spaces, thresholding them, and then combining them to use as image masks. The overall segmentation was carried out following the plant phenotyping procedure described in the documentation of PlantCV v.2 (Figure 2; Gehan et al., 2017). According to this procedure, RGB (Red, Green, Blue) images were first converted to the HSV (Hue, Saturation and Value) color space. The saturation channel was thresholded at 85 to retain as much plant area as possible. A median blur was applied to the binary image to remove background noise. The original image was then converted to LAB color space, and the blue-yellow channel was thresholded again with threshold value 110. Then, a binary mask was created by joining the two binary images to exclude as much background as possible with thresholding without leaving out plant material. The joined binary image was applied as an image mask over the original image. The masked green-magenta and blue-yellow channels were extracted. Then, these two channels were thresholded with threshold values 122 and 110 respectively to capture different portions of the plant. These binary images were then joined together, and small objects were filled. The resulting binary image then served as the image mask to extract the plant area from the binary image.

Next, a rectangular region of interest was defined to retain all image features within this specified region, or to cut the objects to the shape of the region of interest. The position, height, and width of the rectangle was adjusted according to the growth stage of the plant. All isolated objects within this region of interest should now have been of the plant itself. There can be more than one disconnected object that makes up a plant since sometimes leaves twist, making them appear to be separate objects in images. All objects within the defined regions of interest were therefore considered as a single object before further analysis and trait extraction.

Although both the top view and side view images were segmented following the same procedure, some threshold values are different for top and side views segmentations which was ultimately adjusted and finalised by several manual trials. For example, for the side view segmentation, the initial saturation channel was thresholded at 50 before DAS 27, and at 75 after that since plants in the early growing stages were otherwise often covered by the pot rim. All remaining threshold values for side view image segmentation are described in Figure S1.1 which outlines the segmentation steps of a side-view image.


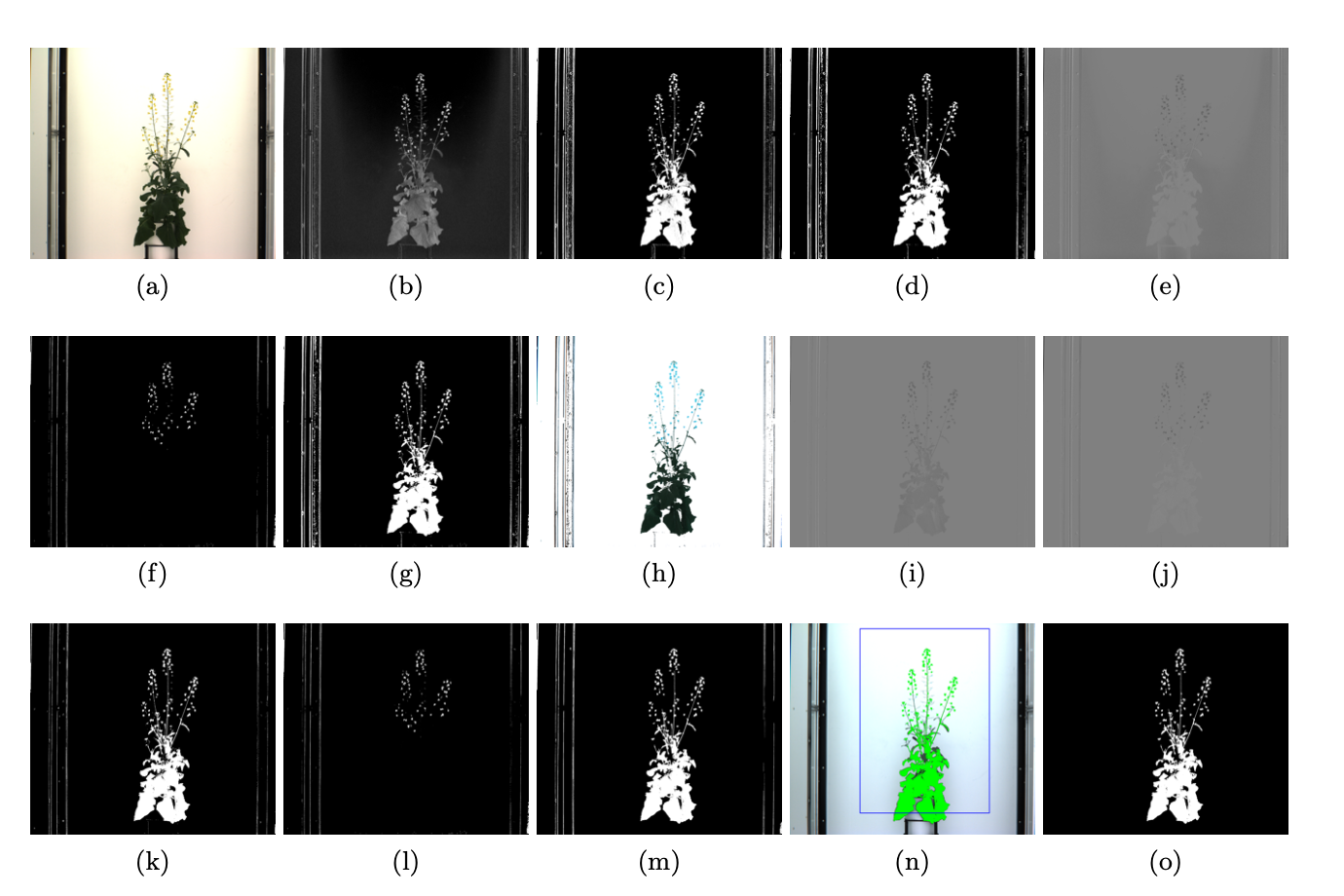


Figure S1.1: (a) The RGB image of a plant at DAS 53 captured from the 0 degree side view. (b) Saturation channel from original RGB image converted to HSV color space. (c) Thresholded saturation channel with threshold value 75. (d) Thresholded saturation channel image with median blur. (e) Blue-yellow channel from LAB color space from original image. (f) Thresholded blue-yellow channel image with threshold value 110. (g) Joined binary images from (d) and (f). (h) Masked image with background removed. (i) The green-magenta channel image. (j) The blue-yellow channel image. (k) Thresholded green-magenta channel image with threshold value 125. (l) Thresholded blue-yellow channel image with threshold value 110. (m) Combined thresholded images. (n) The rectangular region of interest is defined. (o) The final segmented plant area.

1. **Semi-automated flower detection:**

Semi-automated flower detection was performed on the segmented top view images and on their corresponding thresholded blue-yellow channel images for DAS 34 (no plants flowered before this day) to DAS 59 (the last day of the experiment). First, the segmented plant area from top view images were used as image masks (Fig. S1.1f) on the thresholded blue-yellow channel image (Fig. S3.1g) to detect all flower pixels. The resulting masked thresholded blue-yellow channel images were thresholded again to distinguish between actual flowers and presence of flower artefacts (e.g., dried petals). This threshold value was obtained using a treatment-group-specific pixel-threshold based on the overall number of pixels in the masked thresholded blue-yellow channel on each phenotyped time point. For example, on DAS 55, plants of the control group that had more than 965 pixels in the masked thresholded blue yellow channel images were counted as flowering, while plants of the treatment group were only counted as flowering if they had more than 1425 pixels in the masked thresholded blue-yellow channel images. These threshold values were decided after inspecting the number of pixels in the masked thresholded blue-yellow channel images. This was done because the treatment groups exhibited drastic differences in the number of flowering individuals and corresponding flower pixels at some phenotyping time points, in particular at the beginning of the flowering period (Fig. 3).

In order to quantify the onset of flowering (anthesis), we first calculated the bounding box of the segmented plant area for all images. Considering all the flowering plants flowered at least after DAS 34, we labeled all the images as not flowering that had less than 50 pixels within that bounding box area in the masked thresholded blue-yellow channel image. Then, we carried out second level filtering by labelling all the images as not flowering that had less than 25 pixels within the plant boundary in the masked thresholded blue-yellow channel image, for example Figure S1.2c. Then, the images were thresholded again with the concrete number of pixels used as a threshold depending on plant ages and treatment groups. Figure S1.3 shows an example of the start of flowering in a control plant (not flowering on DAS 44, Fig. S1.2a, then flowering on DAS 45 , Fig S1.2e). These threshold values were picked by analyzing the number of pixels in the masked thresholded blue-yellow channel of all flowering and not flowering plant images.


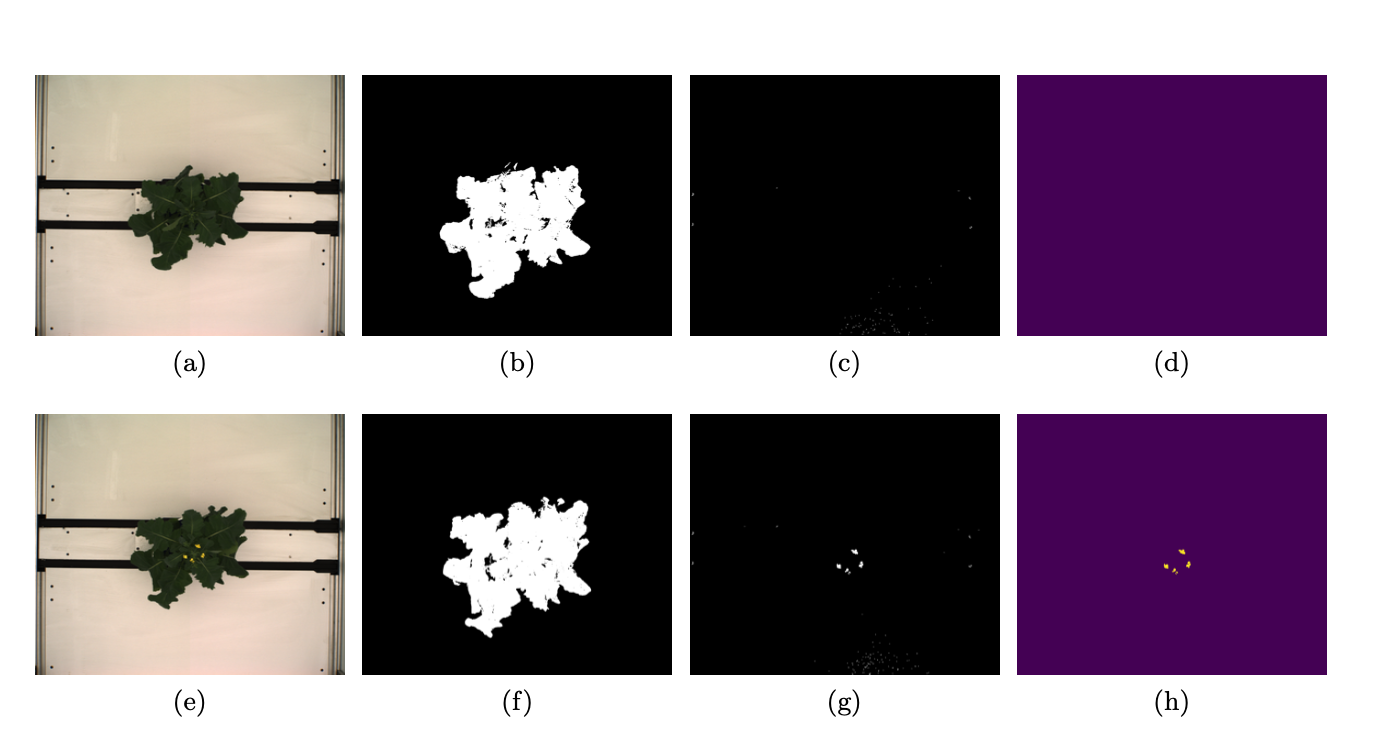


Figure S1.2: (a) The RGB image of a control plant at DAS 44 captured from top view. (b) The segmented plant area of Figure S1.2a. (c) The masked thresholded blue-yellow channel image. (d) The masked thresholded blue-yellow channel image that has less than 25 pixels in the plant area. (e) The RGB image of the same control plant captured the next day from top view. (f) The segmented plant area of Figure S1.2e. (g) The masked thresholded blue-yellow channel image. (h) The isolated flower pixels.


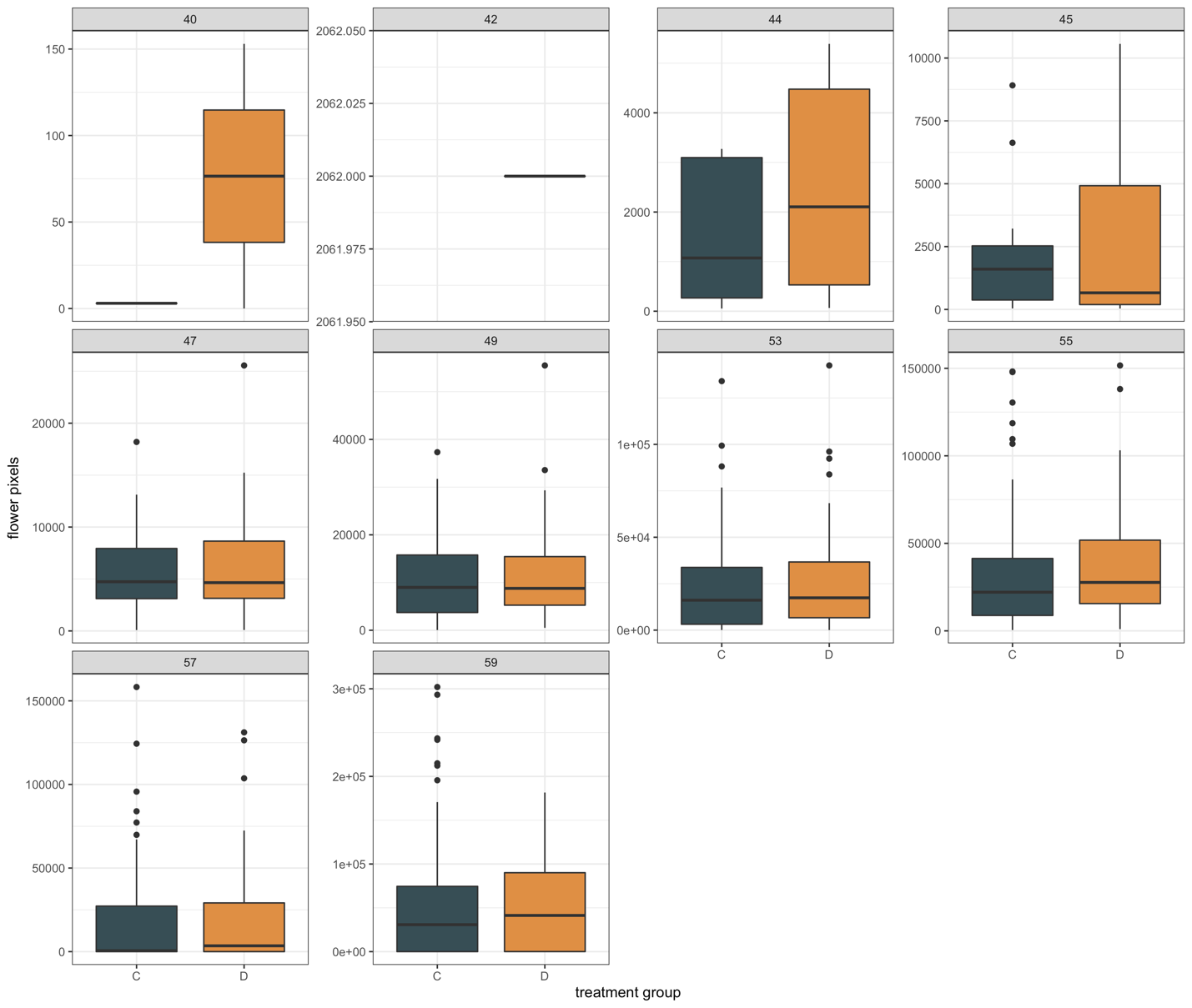


Figure S1.3: Number of flower pixels per phenotyping time point and treatment group.

1. **Flower and raceme branch quantification**

Following successful identification of flowering individuals and onset of flowering, flower output was quantified via quantification of individual components present in the masked thresholded blue-yellow channel images (e.g., Fig. S3.2e). Components were considered to be either single flowers or a number of overlapping flowers and component properties (number of pixels, minimum and maximum x, y coordinate of the bounding box of a component, and the bounding box’s area, convex-hull area, and centroid of each component were extracted). Very small components of less than 100 pixels, likely presenting artefacts (e.g., dried petals) were removed from the dataset while large components were divided by the number of pixels of one fully opened flower seen from the top (700, see 2_NAM_428 on 2019-02-20) to obtain an estimate of the number of overlapping flowers.

Then, hierarchical clustering was applied on all of the above mentioned features to attribute the individual flower components to a cluster, which are predicted to represent inflorescence branches (Figure 4D). Hierarchical clustering is one of the most common forms of unsupervised learning to draw inferences from unlabelled data. The algorithms’ goal is to create clusters that are coherent internally, but clearly different from each other externally. In other words, entities within a cluster should be as similar as possible and entities in one cluster should be as dissimilar as possible from entities in other clusters (reference). The filtered object counts were then used in hierarchical clustering algorithm to assign individual flowers to individual inflorescence branches based on minimum and maximum x and y coordinates and as well as object centroid coordinates. Object assignment was checked visually on a random subsample of 75 images. Then, cluster centroids were calculated in order to estimate the average distance between raceme branches, and maximum canopy diameter and canopy angle were calculated to further describe canopy architecture.

Importantly, we tested having the total number of clusters being determined by the clustering algorithm itself (using the elbow method). In clustering analysis, the elbow method is a heuristic method that is used to determine the number of clusters in a data set. This method consists of plotting the explained variation as a function of the number of clusters, and then picking the elbow of the curve as the number of clusters to use. However, we found that this method did not lead to appropriate cluster numbers and thus also led to misassignment of the flower components to raceme branches. As an example, for Figure 4D, the elbow method returned n = 6 clusters, but the actual number of raceme branches was 12. For this reason, the output from our supervised machine learning algorithms were used to determine the number of raceme branches for each plant and phenotyping time point as an input to the hierarchical clustering.
