# Supplementary material for: Exploiting High-Throughput Indoor Phenotyping to Characterize the Founders of a Structured B. napus Breeding Population
Source: Front Plant Sci. 2022 Jan 5;12:780250. doi: 10.3389/fpls.2021.780250 (PMC8767643; doi:10.3389/fpls.2021.780250)
Supplement: Supplementary file 3 [file Data_Sheet_3.docx]

**Appendix S3: Supplementary Figures and Tables**

Tab S3.1: Sample IDs, descriptions, morphotypes, origins and eigenvectors of the first two principal components for 297 *Brassica napus* accessions included in Principal Component Analysis.

| **Sample ID** | **description** | **morphotype** | **origin** | **PC1** | **PC2** |
| --- | --- | --- | --- | --- | --- |
| DH12075 | Spring type | S | Canada | -0.084452 | 0.022533 |
| Westar | Spring type | S | Canada | -0.097027 | 0.116842 |
| Quantum | Spring type | S | Canada | -0.059782 | 0.021063 |
| Kinki 28 SRS 3704 #1 | Japan Spring type | S | Asia | -0.084224 | 0.027201 |
| Dong Hae 3626 #1 | Korea Spring type | S | Asia | 0.068473 | -0.014812 |
| Kuju 19 SRS 3722 #2 | Korea Spring type | S | Asia | 0.048515 | -0.104675 |
| Buk Wuk 16 SRS 3611 #1 | Korea Spring type | S | Asia | -03687 | -0.011147 |
| PSA12 | Resynthesized | RS | Resynthesised | -0.064174 | -0.095242 |
| N00-C155 | Spring type | S | unknown | -0.050833 | -0.078209 |
| Azuma 22 SRS 3596 #1 | Korea Spring type | S | Asia | -0.021416 | 07082 |
| No Name srs 1631 #2 | Pakistan Spring type | S | Asia | -03035 | -0.151861 |
| Buk Wuk 7 SRS 3606 #1 | Korea Spring type | S | Asia | 0.051361 | -0.049460 |
| Tower CN 33082 #1 | Spring type | S | Canada | -0.120412 | 0.070569 |
| YN01-429 | Yellow seed napus | NA | NA | -0.055606 | 0.086410 |
| Azuma 156 SRS 3597 #1 | Korea Spring type | S | Asia | 0.031632 | -0.109820 |
| Tokiwa natane #1 | Japan Spring type | S | Asia | 0.079571 | 04085 |
| Kuju 41 SRS 3736 #1 | Korea Spring type | S | Asia | -0.048481 | 0.023024 |
| Abukuma natane CN 31408 #1 | Japan Spring type | S | Asia | -0.025631 | 04278 |
| Yudal | Spring type | S | Europe | 0.038385 | -0.049919 |
| Stellar | Spring type | S | Canada | -0.119110 | 0.072963 |
| N99-508 | Spring type | S | Canada | -0.100162 | 0.040084 |
| Dae Chosen SRS 1721 #1 | Korea Spring type | S | Asia | 0.068149 | -0.055618 |
| Mo 83-5 #2 | China Spring type | S | Asia | 0.011820 | -0.128335 |
| Dong Hae 19 SRS 3640 #2 | Korea Spring type | S | Asia | 0.075615 | -06895 |
| Kinki 30 SRS 3706 #1 | Japan Spring type | S | Asia | 0.053876 | -0.049837 |
| Topas | Spring type | S | Canada | -0.088000 | 0.099222 |
| Scoop | Spring type | S | Australia | -08370 | 0.042251 |
| Dong Hae 9 1500200098 #2 | Korea Spring type | S | Asia | -0.059844 | -0.035761 |
| Kinki SRS 3700 #1 | Japan Spring type | S | Asia | 0.076490 | -06880 |
| Buk Wuk 27 SRS 3618 #2 | Korea Spring type | S | Asia | -0.030388 | 0.013410 |
| Polo | Spring type | S | Canada | -0.131429 | 0.060098 |
| Pak 85487 #2 | Pakistan spring type | S | Asia | -0.045182 | -0.094708 |
| No Name SRS 1630 #1 | Pakistan spring type | S | Asia | -0.016989 | -0.114225 |
| Buk Wuk 14 SRS 3609 #1 | Korea spring type | S | Asia | -08336 | 0.042368 |
| Dong Buk SRS 3625 #1 | Korea spring type | S | Asia | 0.022479 | -0.149797 |
| Surpass | Spring type | S | Australia | -0.103779 | 0.041739 |
| Mendel | Winter type | S | Europe | -08655 | -0.159075 |
| Rainbow | Spring type | S | Australia | 02105 | -0.015847 |
| PAK 85484 CN 101858 #2 | Pakistan spring type | S | Asia | -0.012368 | -0.151132 |
| Kuju 57 SRS 1726 #1 | Korea spring type | S | Asia | 0.047198 | -0.060721 |
| Qingyou 4 SRS 1966 #3 | China spring type | S | Asia | 0.081886 | -0.010147 |
| Dong Hae 12 SRS 3635 #1 | Korea spring type | S | Asia | 0.052086 | -0.050137 |
| No Name SRS 471 #2 | China Spring type | S | Asia | 0.080828 | 08729 |
| No Name SRS 1975 #1 | China Spring type | S | Asia | 06071 | 0.059985 |
| Dong Hae 1 SRS 3627 #2 | Korea Spring type | S | Asia | 0.025145 | 0.033566 |
| Kinki Wase SRS 3707 #2 | Japan Spring type | S | Asia | 0.042636 | -0.045463 |
| Kuju 29 SRS 3728 #1 | Korea Spring type | S | Asia | 0.020960 | -0.050656 |
| N | HAU (Ningyou7) | sW | Chinese | 0.064988 | -09246 |
| S2 | HAU | S | Asia | -0.072982 | 0.010071 |
| S3 | HAU | S | Asia | -0.059822 | -0.088225 |
| S4 | HAU (Ny-1) | sW | Chinese | 0.084717 | -09482 |
| S5 | HAU | S | Asia | -0.059025 | 0.042751 |
| S7 | HAU (shuanyou2) | sW | Chinese | 0.085040 | 08879 |
| S8 | HAU (shengliyoucai) | sW | Chinese | 0.048947 | -0.057685 |
| P1 | HAU (6-3476 WT) | sW | Chinese | -00628 | -0.044801 |
| P2 | HAU ( | sW | Chinese | 0.019425 | 0.052185 |
| 8349725010_R01C01 | COSR | sW | Chinese | 0.049050 | 0.027857 |
| 8349725010_R01C02 | COSR | sW | Chinese | 0.027194 | -0.018184 |
| 8349725010_R02C02 | COSR | sW | Chinese | -09129 | 0.011687 |
| 8349725010_R03C01 | COSR | sW | Chinese | 0.034397 | 07226 |
| 8349725010_R03C02 | COSR | sW | Chinese | 0.045046 | 0.043008 |
| 8349725010_R04C01 | COSR | sW | Chinese | 0.021569 | 0.050288 |
| 8349725010_R04C02 | COSR | sW | Chinese | -06248 | -03453 |
| 8349725010_R05C01 | COSR | sW | Chinese | -01532 | -0.080054 |
| 8349725010_R05C02 | COSR | sW | Chinese | 0.032402 | -0.024194 |
| 8349725010_R06C01 | COSR | sW | Chinese | 0.050412 | 0.055314 |
| 8349725010_R06C02 | COSR | sW | Chinese | 0.057476 | 0.040300 |
| 8349725010_R07C01 | COSR | sW | Chinese | 0.055044 | 0.018076 |
| 8349725010_R08C01 | COSR | sW | Chinese | -03299 | 09319 |
| 8349725010_R08C02 | COSR | sW | Chinese | 0.020410 | -03589 |
| 8349725010_R09C01 | COSR | sW | Chinese | 0.052672 | 0.042523 |
| 8349725010_R09C02 | COSR | sW | Chinese | 0.030569 | -03584 |
| 8349725010_R10C01 | COSR | sW | Chinese | 0.060278 | -04009 |
| 8349725010_R10C02 | COSR | sW | Chinese | 0.059886 | 0.037358 |
| 8349725010_R11C01 | COSR | sW | Chinese | 0.029051 | -00745 |
| 8349725010_R11C02 | COSR | sW | Chinese | 0.038419 | -09092 |
| 8349725010_R12C01 | COSR | sW | Chinese | 0.053271 | 0.035321 |
| 8349725010_R12C02 | COSR | sW | Chinese | 0.074704 | 0.026067 |
| 8349725012_R01C01 | COSR | sW | Chinese | 0.045785 | 0.046078 |
| 8349725012_R02C01 | COSR | sW | Chinese | 0.019141 | -0.023696 |
| 8349725012_R02C02 | COSR | sW | Chinese | -0.017639 | -0.069023 |
| 8349725012_R03C01 | COSR | sW | Chinese | 0.018811 | 0.032499 |
| 8349725012_R03C02 | COSR | sW | Chinese | 07538 | -0.069989 |
| 8349725012_R04C02 | COSR | sW | Chinese | 0.033063 | -0.036817 |
| 8349725012_R05C01 | COSR | sW | Chinese | 0.035231 | 0.018221 |
| 8349725012_R05C02 | COSR | sW | Chinese | 03196 | 07505 |
| 8349725012_R06C01 | COSR | sW | Chinese | 0.059835 | 0.029920 |
| 8349725012_R06C02 | COSR | sW | Chinese | 0.068739 | 0.030505 |
| 8349725012_R07C01 | COSR | sW | Chinese | 0.052222 | 0.035613 |
| 8349725012_R07C02 | COSR | sW | Chinese | 0.044872 | 04396 |
| 8349725012_R08C01 | COSR | sW | Chinese | 08161 | -0.014479 |
| 8349725012_R08C02 | COSR | sW | Chinese | 0.048319 | -0.058963 |
| 8349725012_R09C01 | COSR | sW | Chinese | 0.049371 | 0.044350 |
| 8349725012_R09C02 | COSR | sW | Chinese | 0.056499 | 0.034783 |
| 8349725012_R10C02 | COSR | sW | Chinese | 0.052484 | 0.037966 |
| 8349725012_R11C01 | COSR | sW | Chinese | -0.011216 | -0.015182 |
| 8349725012_R11C02 | COSR | sW | Chinese | 0.041359 | -0.033767 |
| 8349725012_R12C01 | COSR | sW | Chinese | 0.041033 | 0.047722 |
| 8349725012_R12C02 | COSR | sW | Chinese | 0.081256 | 0.010548 |
| 8349725016_R01C01 | COSR | sW | Chinese | 02048 | -0.018376 |
| 8349725016_R01C02 | COSR | sW | Chinese | 0.014342 | -0.038259 |
| 8349725016_R02C01 | COSR | sW | Chinese | 09738 | 0.039287 |
| 8349725016_R02C02 | COSR | sW | Chinese | 0.052547 | 0.041067 |
| 8349725016_R03C01 | COSR | sW | Chinese | 0.054155 | 07015 |
| 8349725016_R03C02 | COSR | sW | Chinese | 03114 | 07542 |
| 8349725016_R04C01 | COSR | sW | Chinese | 0.052983 | -0.038720 |
| 8349725016_R04C02 | COSR | sW | Chinese | 0.044488 | 0.036767 |
| 8349725016_R05C01 | COSR | sW | Chinese | -03018 | -0.037438 |
| 8349725016_R05C02 | COSR | sW | Chinese | 0.019685 | 0.017094 |
| 8349725016_R06C01 | COSR | sW | Chinese | 0.057811 | 0.038575 |
| 8349725016_R06C02 | COSR | sW | Chinese | 0.031097 | 0.032162 |
| 8349725016_R07C01 | COSR | sW | Chinese | 0.044779 | 0.013901 |
| 8349725016_R08C01 | COSR | sW | Chinese | 0.054450 | 0.037332 |
| 8349725016_R08C02 | COSR | sW | Chinese | -01393 | 0.021388 |
| 8349725016_R09C01 | COSR | sW | Chinese | 0.052948 | 0.033050 |
| 8349725016_R09C02 | COSR | sW | Chinese | 0.048079 | -01478 |
| 8349725016_R10C01 | COSR | sW | Chinese | 0.018565 | -0.016319 |
| 8349725016_R10C02 | COSR | sW | Chinese | 01174 | 0.070968 |
| 8349725016_R11C01 | COSR | sW | Chinese | 0.042046 | 0.026972 |
| 8349725016_R11C02 | COSR | sW | Chinese | 0.027834 | -0.056905 |
| 8349725016_R12C01 | COSR | sW | Chinese | 0.072223 | 0.010209 |
| 8349725016_R12C02 | COSR | sW | Chinese | 0.076529 | -07048 |
| 8349725023_R01C01 | COSR | sW | Chinese | -08770 | -0.049225 |
| 8349725023_R01C02 | COSR | sW | Chinese | 0.032830 | 0.042556 |
| 8349725023_R02C01 | COSR | sW | Chinese | 0.052541 | 0.041098 |
| 8349725023_R02C02 | COSR | sW | Chinese | 0.036134 | -04059 |
| 8349725023_R03C01 | COSR | sW | Chinese | 0.057681 | 07263 |
| 8349725023_R03C02 | COSR | sW | Chinese | 08761 | -0.023534 |
| 8349725023_R04C01 | COSR | sW | Chinese | 0.053451 | 0.035332 |
| 8349725023_R04C02 | COSR | sW | Chinese | 0.064825 | -0.015638 |
| 8349725023_R05C01 | COSR | sW | Chinese | 0.037736 | 00079 |
| 8349725023_R05C02 | COSR | sW | Chinese | 0.024641 | 0.027254 |
| 8349725023_R06C01 | COSR | sW | Chinese | 0.045855 | 0.028902 |
| 8349725023_R06C02 | COSR | sW | Chinese | 08381 | 0.073234 |
| 8349725023_R07C01 | COSR | sW | Chinese | 0.088924 | 08390 |
| 8349725023_R08C01 | COSR | sW | Chinese | 0.033083 | 06854 |
| 8349725023_R08C02 | COSR | sW | Chinese | 0.054420 | 0.048374 |
| 8349725023_R09C01 | COSR | sW | Chinese | 0.035199 | 0.039692 |
| 8349725023_R10C01 | COSR | sW | Chinese | 0.011617 | 00444 |
| 8349725023_R10C02 | COSR | sW | Chinese | 0.046445 | 0.029799 |
| 8349725023_R11C01 | COSR | sW | Chinese | 0.029549 | -0.025980 |
| 8349725023_R11C02 | COSR | sW | Chinese | 0.041672 | 0.018392 |
| 8349725023_R12C01 | COSR | sW | Chinese | 0.084133 | -09325 |
| 8349725023_R12C02 | COSR | sW | Chinese | 0.074741 | 0.010009 |
| 8349725025_R01C01 | COSR | sW | Chinese | 0.056392 | 0.023798 |
| 8349725025_R01C02 | COSR | sW | Chinese | -0.023146 | -07133 |
| 8349725025_R02C02 | COSR | sW | Chinese | -0.017129 | -00653 |
| 8349725025_R03C01 | COSR | sW | Chinese | -0.013811 | 0.068056 |
| 8349725025_R03C02 | COSR | sW | Chinese | 0.044727 | 0.018847 |
| 8349725025_R04C01 | COSR | sW | Chinese | 0.032823 | -03718 |
| 8349725025_R04C02 | COSR | sW | Chinese | 0.016465 | -0.021776 |
| 8349725025_R06C01 | COSR | sW | Chinese | 0.028048 | 0.014559 |
| 8349725025_R07C02 | COSR | sW | Chinese | 0.045028 | 0.047124 |
| 8349725025_R08C01 | COSR | sW | Chinese | 0.049079 | 0.028160 |
| 8349725025_R09C01 | COSR | sW | Chinese | 0.013004 | -0.016695 |
| 8349725025_R10C01 | COSR | sW | Chinese | 0.044467 | 0.053186 |
| 8349725025_R10C02 | COSR | sW | Chinese | 0.013282 | -0.017072 |
| 8349725025_R11C02 | COSR | sW | Chinese | 0.057650 | 0.047323 |
| 8349725025_R12C01 | COSR | sW | Chinese | 0.017237 | 0.011995 |
| 8349725025_R12C02 | COSR | sW | Chinese | 0.044211 | 0.042126 |
| 8349725027_R01C01 | COSR | sW | Chinese | -0.017450 | -00983 |
| 8349725027_R01C02 | COSR | sW | Chinese | 0.058735 | 0.035551 |
| 8349725027_R02C01 | COSR | sW | Chinese | 0.059551 | 0.049094 |
| 8349725027_R03C01 | COSR | sW | Chinese | 0.028177 | 06785 |
| 8349725027_R03C02 | COSR | sW | Chinese | 0.020443 | 05296 |
| 8349725027_R04C02 | COSR | sW | Chinese | 0.051642 | 0.044640 |
| 8349725027_R06C02 | COSR | sW | Chinese | 0.017853 | -05819 |
| 8349725027_R07C01 | COSR | sW | Chinese | 0.037086 | -09188 |
| 8349725027_R07C02 | COSR | sW | Chinese | 0.048878 | 0.025336 |
| 8349725027_R09C01 | COSR | sW | Chinese | 0.029746 | -0.060530 |
| 8349725027_R09C02 | COSR | sW | Chinese | 0.015376 | -01306 |
| 8349725027_R10C01 | COSR | sW | Chinese | 0.044204 | 0.039308 |
| 8349725027_R10C02 | COSR | sW | Chinese | 0.023854 | -0.034403 |
| 8349725027_R11C01 | COSR | sW | Chinese | 0.027002 | 0.015900 |
| 8349725027_R11C02 | COSR | sW | Chinese | 0.030790 | 0.010045 |
| 8349725027_R12C01 | COSR | sW | Chinese | 0.050968 | 0.033032 |
| 8349725027_R12C02 | COSR | sW | Chinese | 01765 | -0.072996 |
| 8349725058_R01C01 | COSR | sW | Chinese | 0.060812 | 0.011046 |
| 8349725058_R02C02 | COSR | sW | Chinese | 0.033851 | -0.010971 |
| 8349725058_R03C01 | COSR | sW | Chinese | -0.018191 | -0.059414 |
| 8349725058_R03C02 | COSR | sW | Chinese | 0.045584 | 0.039907 |
| 8349725058_R04C01 | COSR | sW | Chinese | 0.044141 | -0.042814 |
| 8349725058_R04C02 | COSR | sW | Chinese | -07715 | 0.058368 |
| 8349725058_R06C01 | COSR | sW | Chinese | 0.010339 | -0.044627 |
| 8349725058_R06C02 | COSR | sW | Chinese | 0.046627 | 0.057190 |
| 8349725058_R07C01 | COSR | sW | Chinese | 0.058179 | 0.039515 |
| 8349725058_R08C01 | COSR | sW | Chinese | 0.038599 | 09887 |
| 8349725058_R08C02 | COSR | sW | Chinese | 0.031162 | -0.044776 |
| 8349725058_R09C01 | COSR | sW | Chinese | 0.049032 | 0.025722 |
| 8349725058_R09C02 | COSR | sW | Chinese | 0.060368 | 0.050759 |
| 8349725058_R10C01 | COSR | sW | Chinese | 0.038399 | 0.035745 |
| 8349725058_R10C02 | COSR | sW | Chinese | 0.041290 | 0.042768 |
| 8349725058_R11C01 | COSR | sW | Chinese | 0.053222 | 0.036753 |
| 8349725058_R11C02 | COSR | sW | Chinese | 0.011551 | -0.012482 |
| 8349725058_R12C01 | COSR | sW | Chinese | 0.050363 | 0.041632 |
| 8349725058_R12C02 | COSR | sW | Chinese | 0.047935 | 0.050974 |
| 8349725059_R01C01 | COSR | sW | Chinese | 01865 | 0.053446 |
| 8349725059_R01C02 | COSR | sW | Chinese | 0.043599 | 0.044769 |
| 8349725059_R03C01 | COSR | sW | Chinese | 0.063055 | 0.027322 |
| 8349725059_R03C02 | COSR | sW | Chinese | -0.019683 | 0.028897 |
| 8349725059_R04C01 | COSR | sW | Chinese | 0.013426 | -0.017023 |
| 8349725059_R05C02 | COSR | sW | Chinese | 0.050449 | 0.015965 |
| 8349725059_R06C01 | COSR | sW | Chinese | 0.037981 | 0.010867 |
| 8349725059_R06C02 | COSR | sW | Chinese | 0.037262 | 07687 |
| 8349725059_R07C01 | COSR | sW | Chinese | 0.013354 | 00947 |
| 8349725059_R08C01 | COSR | sW | Chinese | -02989 | -0.074614 |
| 8349725059_R08C02 | COSR | sW | Chinese | 0.033883 | 0.026432 |
| 8349725059_R09C01 | COSR | sW | Chinese | 0.058094 | 0.038998 |
| 8349725059_R10C01 | COSR | sW | Chinese | 0.059193 | 0.050738 |
| 8349725059_R11C01 | COSR | sW | Chinese | 0.052114 | 0.037496 |
| 8349725059_R11C02 | COSR | sW | Chinese | 0.056605 | 0.046753 |
| 8349725059_R12C01 | COSR | sW | Chinese | 0.026928 | 0.054995 |
| 8349725059_R12C02 | COSR | sW | Chinese | 0.044714 | 0.051836 |
| Ag-Spectrum | Ag-Spectrum | S | Australia | -0.079008 | -0.087632 |
| Dunkeld | Dunkeld | S | Australia | -0.073294 | -0.050198 |
| Argyle | Argyle | S | Australia | -0.088880 | -0.067035 |
| Nurendra | Nurendra | S | Australia | -0.089454 | -0.068522 |
| ATR_Cobbler | ATR_Cobbler | S | Australia | -0.085683 | -0.073507 |
| RSO | RSO | S | unknown | -0.081695 | -0.072148 |
| ATR-Stingray | ATR-Stingray | S | Australia | -0.075393 | -0.052064 |
| Skipton | Skipton | S | Australia | -0.072875 | -0.054242 |
| ATR-Summit | ATR-Summit | S | Australia | -0.071346 | -0.064577 |
| Taparoo | Taparoo | S | Australia | -0.078518 | -0.040488 |
| AV-Sapphire | AV-Sapphire | S | Australia | -0.079022 | -0.045168 |
| Telfer | Telfer | S | Australia | -0.083033 | -0.070045 |
| Trilogy | Trilogy | S | Australia | -0.084993 | 04186 |
| Bravo-TT | Bravo-TT | S | Australia | -0.077222 | -0.042711 |
| Thunder-TT | Thunder-TT | S | Australia | -0.068184 | -0.057451 |
| Carousel | Carousel | S | Australia | -0.080450 | -0.046213 |
| Tornado-TT | Tornado-TT | S | Australia | -0.069007 | -0.056376 |
| Surpass400_024DH | Surpass400_024DH | S | Australia | -0.034214 | -02581 |
| Monty_028DH | Monty_028DH | S | Australia | 0.090635 | 03133 |
| NuSeed 1 | NuSeed 1 | S | Australia | -0.052315 | 0.050295 |
| NuSeed 7 | NuSeed 7 | S | Australia | -0.052641 | 0.058223 |
| NuSeed 2 | NuSeed 2 | S | Australia | -0.042108 | 0.047474 |
| NuSeed 8 | NuSeed 8 | S | Australia | -0.040607 | 0.046714 |
| NuSeed 3 | NuSeed 3 | S | Australia | -0.049540 | 0.049702 |
| NuSeed 9 | NuSeed 9 | S | Australia | -0.043129 | 0.049734 |
| NuSeed 4 | NuSeed 4 | S | Australia | -0.037453 | 0.046823 |
| NuSeed 10 | NuSeed 10 | S | Australia | -0.046480 | 0.054873 |
| NuSeed 5 | NuSeed 5 | S | Australia | -0.062591 | -05922 |
| NuSeed 6 | NuSeed 6 | S | Australia | -0.048474 | 0.058502 |
| NAM_1_A | SKBnNAM founder | NAM | Europe | -0.060451 | 0.082907 |
| NAM_4_A | SKBnNAM founder | NAM | Australia | -0.035700 | -0.020977 |
| NAM_5_A | SKBnNAM founder | NAM | Asia | -0.092870 | 0.037572 |
| NAM_8_A | SKBnNAM founder | NAM | unknown | -07628 | 0.064581 |
| NAM_10_A | SKBnNAM founder | NAM | Europe | -0.097027 | 0.050617 |
| NAM_12_A | SKBnNAM founder | NAM | Canada | -0.078457 | 0.035901 |
| NAM_13_A | SKBnNAM founder | NAM | Europe | -0.115069 | 0.059128 |
| NAM_14_A | SKBnNAM founder | NAM | Europe | -0.088636 | -0.037973 |
| NAM_15_A | SKBnNAM founder | NAM | Australia | -0.087444 | 0.051290 |
| NAM_17_A | SKBnNAM founder | NAM | Australia | -0.056866 | 0.017581 |
| NAM_23_A | SKBnNAM founder | NAM | Asia | -0.090722 | 0.104803 |
| NAM_25_A | SKBnNAM founder | NAM | Argentina | -0.071286 | 0.088294 |
| NAM_26_A | SKBnNAM founder | NAM | Argentina | -0.097935 | 0.041783 |
| NAM_28_A | SKBnNAM founder | NAM | Europe | -0.083629 | 0.084483 |
| NAM_29_A | SKBnNAM founder | NAM | Resynthesised | -0.065517 | -0.096259 |
| NAM_30_A | SKBnNAM founder | NAM | Europe | -0.076600 | 0.028464 |
| NAM_31_A | SKBnNAM founder | NAM | Asia | -0.050799 | 07617 |
| NAM_32_A | SKBnNAM founder | NAM | Asia | -0.042689 | 0.017793 |
| NAM_33_A | SKBnNAM founder | NAM | Asia | -0.090166 | -0.040500 |
| NAM_34_A | SKBnNAM founder | NAM | Asia | -0.018351 | 0.073864 |
| NAM_36_A | SKBnNAM founder | NAM | unknown | -0.074061 | 0.011074 |
| NAM_37_A | SKBnNAM founder | NAM | Australia | -0.090000 | 0.045233 |
| NAM_38_A | SKBnNAM founder | NAM | Australia | -0.102788 | 0.047671 |
| NAM_39_A | SKBnNAM founder | NAM | Asia | -0.057276 | -0.016541 |
| NAM_40_A | SKBnNAM founder | NAM | Europe | -0.089136 | 0.044262 |
| NAM_43_A | SKBnNAM founder | NAM | Asia | -0.062105 | 02377 |
| NAM_0 | SKBnNAM founder, common parent | NAM | Canada | -0.100141 | 0.040191 |
| NAM_51A | SKBnNAM founder | NAM | Canada | -0.066329 | -00957 |
| NAM_53A | SKBnNAM founder | NAM | Asia | -0.038609 | 0.012795 |
| NAM_56A | SKBnNAM founder | NAM | Europe | -0.091622 | 0.044478 |
| NAM_57A | SKBnNAM founder | NAM | Europe | -0.086501 | -0.032398 |
| NAM_65A | SKBnNAM founder | NAM | Europe | -0.075277 | -0.052280 |
| NAM_66A | SKBnNAM founder | NAM | unknown | 0.031600 | -0.061290 |
| NAM_68A | SKBnNAM founder | NAM | Europe | -0.114056 | 0.058182 |
| NAM_71A | SKBnNAM founder | NAM | Canada | -0.107031 | 0.064907 |
| NAM_72A | SKBnNAM founder | NAM | Canada | -0.051433 | -04204 |
| NAM_73A | SKBnNAM founder | NAM | Europe | -0.068934 | 0.084668 |
| NAM_75A | SKBnNAM founder | NAM | unknown | -0.061936 | 0.075381 |
| NAM_76A | SKBnNAM founder | NAM | Canada | -0.066886 | 04808 |
| NAM_78A | SKBnNAM founder | NAM | Canada | -0.071401 | 0.106118 |
| NAM_79A | SKBnNAM founder | NAM | Asia | -0.028034 | -0.067030 |
| NAM_82A | SKBnNAM founder | NAM | Australia | -0.086404 | 0.035010 |
| NAM_83A | SKBnNAM founder | NAM | Asia | 0.068787 | 06253 |
| NAM_85 | SKBnNAM founder | NAM | Asia | 0.068273 | -0.055548 |
| NAM_86 | SKBnNAM founder | NAM | Asia | 0.082265 | -04022 |
| NAM_87 | SKBnNAM founder | NAM | Europe | -0.039662 | -0.037120 |
| NAM_88 | SKBnNAM founder | NAM | Europe | -0.073992 | 0.010013 |
| NAM 42 BULK | SKBnNAM founder | NAM | Asia | -0.087257 | 0.029518 |
| NAM 45 BULK | SKBnNAM founder | NAM | Asia | 03111 | 0.060549 |
| NAM 46 BULK | SKBnNAM founder | NAM | Asia | 0.035027 | -0.105603 |
| NAM 47 BULK | SKBnNAM founder | NAM | Asia | 0.067986 | -0.024886 |
| Svalofs_Gulle | - | S | Europe | -0.088720 | -0.038148 |
| Tribune | - | S | Australia | -0.086531 | 0.035223 |
| ACS_Resyn | - | S | Canada | -0.081121 | 0.106903 |
| Maris_Haplona | - | S | Europe | -0.103565 | 0.048547 |

**Supplementary Tables**

Table S3.2: Detailed information for 51 spring type *B. napus* lines used as founders for SKBnNAM population.

| **NAM CODE** | **Line Name** | **Other Name / Code** | **Other Identifier / Source** | **Pedigree and/or Origin** | **Type** | **2015 gluc** | **2015 22:1** | **Days to flower (2015 field data)** |
| --- | --- | --- | --- | --- | --- | --- | --- | --- |
| NAM-0 | N99-508 | ACS N33 |  | Quantum/LG3260 | Canadian Adapted | 10.04 | 0.36 | 43.1 |
| NAM-1 | Czyzowska | N12-C12616 | BnASSYST-321 | Poland | European | 101.88 | 47.43 | 44.8 |
| NAM-4 | Wesway |  | BnASSYST-285 | Ramses/Oro, Australia | Australian | 86.37 | 0.18 | 40.8 |
| NAM-5 | BN-1 | N11-C11183 | TO10-17106-2 | Tata Energy Research Institute, New Delhi, India | Exotic | 48.36 | 6.69 | 40.9 |
| NAM-8 | 86004 | N11-C11137 | TO10-16792-1,-5 | Unknown | Exotic | 21.55 | 0.25 | 53.5 |
| NAM-10 | Global | N11-C11155 | TO10-16804-4 | **Sv701034 x Sv67613** or Sv701034 x Baldo (Sv02279), Sweden or Denmark | European | 9.44 | 0.26 | 52 |
| NAM-12 | 46A65 |  | BH12-17952-4 | Canadian, Pioneer Hi-Bred Production Limited (Patel) Tested as NS1565 | Canadian Adapted | 19.66 | 0.42 | 43.9 |
| NAM-13 | Campino |  | BnASSYST-291 | Germany | European | 11.05 | 0.2 | 43.7 |
| NAM-14 | Svalöf’s Gulle | N12-C12593 | BnASSYST-282 | Gulzower/Regina x Lembkes/?, Sweden | European | 98.41 | 36.42 | 51.1 |
| NAM-15 | N01D-1330 | N12-C12619 | BnASSYST-258 | Australia | Australian | 9.96 | 0.2 | 47.8 |
| NAM-17 | N00-C3661sp2 | N05-C468 |  | N89-53/Dunkeld//N89-53/Range | Canadian Adapted | 11.08 | 0.36 | 47.8 |
| NAM-23 | Daichousen (mizuyasu) | N12-C12578 | BnASSYST-323 | North Korea | Exotic | 11.57 | 0.41 | 46.3 |
| NAM-25 | Nolza 541 |  | SRS 2629 | Argentina | Other | 15.62 | 0.41 | 43 |
| NAM-26 | Nolza 531 |  | SRS 2630 | Argentina | Other | 14.86 | 0.21 | 51.8 |
| NAM-28 | Topas | N12-C12594 | BnASSYST-283 | Hermes/Bronowski x Gulle/, Sweden | European | 10.62 | 0.31 | 46.4 |
| NAM-29 | PSA12 |  |  |  | Other | 105.56 | 17.24 | 45.7 |
| NAM-30 | Egra | N11-C11159, N12-12509 | TO10-16810-1,-3,-5 | Unknown | European | 10.17 | 0.37 | 50 |
| NAM-31 | Wase Chousen |  | BnASSYST-389 | Korea | Exotic | 23.08 | 0.78 | 51.3 |
| NAM-32 | Dong Hae 2 | SN50 | SRS 3628 | South Korea | Exotic | 122.82 | 0.3 | 43.5 |
| NAM-33 | Dong Hae 3 | SN53 | SRS 3629 | South Korea | Exotic | 101.66 | 19.95 | 51.5 |
| NAM-34 | Dong Hae 11 | SN18 | SRS 3634 | South Korea | Exotic | 116.14 | 21.84 | 47.1 |
| NAM-36 | 81N058-5 | N12-C11629 | TO10-16786-3 | Unknown | Exotic | 83.43 | 4.83 | 47.8 |
| NAM-37 | Wesroona |  | BnASSYST-393 | Norin20/Tower, Australia | Australian | 61.27 | 0.32 | 50.3 |
| NAM-38 | Wesreo |  | BnASSYST-392 | Major/Oro, Australia | Australian | 70.96 | 0.3 | 49.3 |
| NAM-39 | Nakate Chousen |  | BnASSYST-357 | Korea | Exotic | 124.58 | 0.54 | 51.8 |
| NAM-40 | Mlochowski |  | BnASSYST-355 | Pedigree: 'Local', Poland | European | 81.05 | 38.92 | 55.6 |
| NAM-42 | PI432392 | Bau-M/50 | BAUM50 | Bangladesh Ag Univ, Mymensingh, Dept of Gen & Plant Breeding; Donated to USDA Dec-1978 by Rahman, L., Brassica Breeding Project. | Other | 72.06 | 0.25 | 52.2 |
| NAM-43 | PI432395 | Bau-M/71 | BAUM71 | Bangladesh Ag Univ, Mymensingh, Dept of Gen & Plant Breeding; Donated to USDA Dec-1978 by Rahman, L., Brassica Breeding Project. | Other | 60.96 | 8.84 | 44.7 |
| NAM-45 | Dong Hae 6 | PI469765 |  | South Korea | Exotic | 115.91 | 3.73 | 51.9 |
| NAM-46 | Dong Hae 21 | PI469776 |  | South Korea | Exotic | 103.53 | 43.93 | 54.5 |
| NAM-47 | Dong Hae 23 | PI469778 |  | South Korea | Exotic | 105.69 | 44.94 | 57.1 |
| NAM-51 | N12-C12541 | AC Elect | ACS N08; BnASSYST-482 | AAFC Saskatoon Topas/Westar,Karat/Westar,Rabo/Westar (Rakow) | Canadian Adapted | 18.52 | 0.26 | 42.7 |
| NAM-53 | Buk Wuk 27 (-1) | Buk Wuk 27 | SRS 3618 | South Korea | Exotic | 106.41 | 32.3 | 52.7 |
| NAM-56 | BH12-18521-1 | MAZOWIECKI | PI311730; BnASSYST-268 | Pedigree: 'Local', Poland | European | 78.45 | 34.24 | 55.3 |
| NAM-57 | BH12-18548-1 | Mozart | BnASSYST-302 | Denmark | European | 12.74 | 0.36 | 51.6 |
| NAM-65 | N11-11101 | Puma | TO10-16624-1,-5 | Seln from /Sv751406 x Sv74465/, Sweden or Denmark | European | 14.89 | 0.36 | 48.2 |
| NAM-66 | N12-C11609 | 88-23037-3 |  | Unknown | Exotic | 99.93 | 1.39 | 54.7 |
| NAM-68 | BH12-18586-1 | Lirawell; N12-C12615 | BnASSYST-347 | Germany | European | 16.05 | 0.39 | 50.9 |
| NAM-71 | BH12-18031-2 | ACS N22; SRN90-2700 | BnASSYST-477 | Sclerotinia resist Norin11/Regent//Westar (Woods and Verma,1980's) | Canadian Adapted | 17.93 | 0.52 | 49.7 |
| NAM-72 | BH12-18046-1 | ACS YN03-C656 | BnASSYST-493 | Express/3/N93-P1526////Rsyn2-11 [N93-P1526=N89-53/Shiralee//N89-53] Yel sum ann winter | Winter Background | 10.46 | 0.36 | 52.6 |
| NAM-73 | BH12-18596-1 | Optima | BnASSYST-362 | Line x Tower (DP19 29), Denmark | European | 91.34 | 0.13 | 47.9 |
| NAM-75 | BH12-17966-1 | Magnum |  | Limagrain Tested as PRO3096, Canada or Sweden | Canadian Adapted | 11.12 | 0.21 | 43.6 |
| NAM-76 | BH12-17967-2 | Ebony |  | Limagrain Tested as PR2613, Canada | Canadian Adapted | 9.59 | 0.19 | 47.1 |
| NAM-78 | BH12-18059-1 | N12-C12564 | ACS YN06-C0846; BnASSYST-508 | N89-53/YN9592 | Canadian Adapted | 16.02 | 0.28 | 48.8 |
| NAM-79 | PAK 85912 | SN14 | CN 101870 | Pakistan | Exotic | 114.3 | 37.98 | 42.5 |
| NAM-82 | BH12-18551-2 | Tribune | BnASSYST-307; N12-C12611 | Karoo/Surpass400, Australian | Australian | 9.15 | 0.54 | 45.7 |
| NAM-83 | BH12-18498-1 | SWU Chinese 9 | BnASSYST-236; N12-C12574 | China | Exotic | 109.36 | 45.32 | 56.8 |
| NAM-85 | DC21 | SRS 3624 or SRS 1721? | Dae Chosen | South Korea? | Exotic | 78.19 | 43.4 | 61.2 |
| NAM-86 | Kinki 22 | SRS 3703 |  | South Korea | Exotic | 80.9 | 39.11 | 66.8 |
| NAM-87 | Tanto | SRS 151 |  | France | European | 18 | 0.27 | 49.8 |
| NAM-88 | SRS1632 |  |  | Poland | Other | 15.88 | 8.35 | 55.3 |

Table S3.3: Additional details for growing conditions for *B. napus* indoor phenotyping experiment at LemnaTec Scanalyzer 3D facility at University of Nebraska, Lincoln campus

| **Parameter** | **Value** |
| --- | --- |
| Air temperature | 18-22°C |
| Average length of the light period | 16 h |
| Type of lamps used | lumigrow pro 650 LED |
| Atmospheric CO2 concentration | uncontrolled |
| Average relative humidity during the light period | 40 – 60%,: |
| Average relative humidity during the dark period | 40 – 60%,: |
| Rooting medium/potting soil | ProMix BX with osmocote added |
| Container type | pot |
| Container volume | 9L |
| Container height | 260 mm |
| Plants per container | 1 |
| Type and amount of fertiliser added per container/m2 | Osmocote time release / 4 pounds per yard |

Table S3.4: Differences in mean area under the ROC curve (ROC AUC), and mean accuracies for stress identification on DAS 49 under varying input feature combinations. Four combinations were tested in columns two through five, with column two using all features (mean and 75^th^ percentile NIR, ∆ pixels, convex hull area, total number of plant pixels, ∆ convex hull, plant height and width, ∆ plant height, ∆ plant width, ExG, and number of raceme branches), column three using all features except for mean excess green (ExG), column four using all features except for number of raceme branches, and column five using all features except for number of raceme branches and difference in height compared to previous phenotyping time point (*∆* height).

|  | **Mean ROC AUC (Mean accuracy with 5-fold cross validation (SD))** | | | |
| --- | --- | --- | --- | --- |
|  | All | All - ExG | All - branches | All - branches -  ∆ height |
| **Random Forest** | **0.85(0.81(±0.03))** | 0.84(0.77(±0.10)) | 0.84(0.79(±0.11)) | 0.85(0.75(±0.08)) |
| **Linear Discriminant Analysis** | 0.82(0.77(±0.04)) | 0.82(0.71(±0.16)) | 0.82(0.70 (±0.14)) | 0.83(0.72 (±0.12)) |
| **Logistic Regression** | 0.82(0.79(±0.08)) | 0.82(0.70(±0.21)) | 0.82(0.75 (±0.17)) | 0.81(0.76(±0.14)) |
| **Decision Tree** | 0.73(0.75(±0.12)) | 0.72(0.76(±0.12)) | 0.74(0.73 (±0.11)) | 0.76(0.76(±0.14)) |
| **KNN classifier (K=1)** | 0.72(0.73(±0.08)) | 0.73(0.73(±0.08)) | 0.73(0.73((±0.08)) | 0.73(0.73 (±0.08)) |
| **Support Vector Machine** | 0.76(0.67(±0.23)) | 0.76(0.67(±0.22)) | 0.76(0.67 (±0.22)) | 0.76(0.67 (±0.22)) |

**Supplementary Figures**


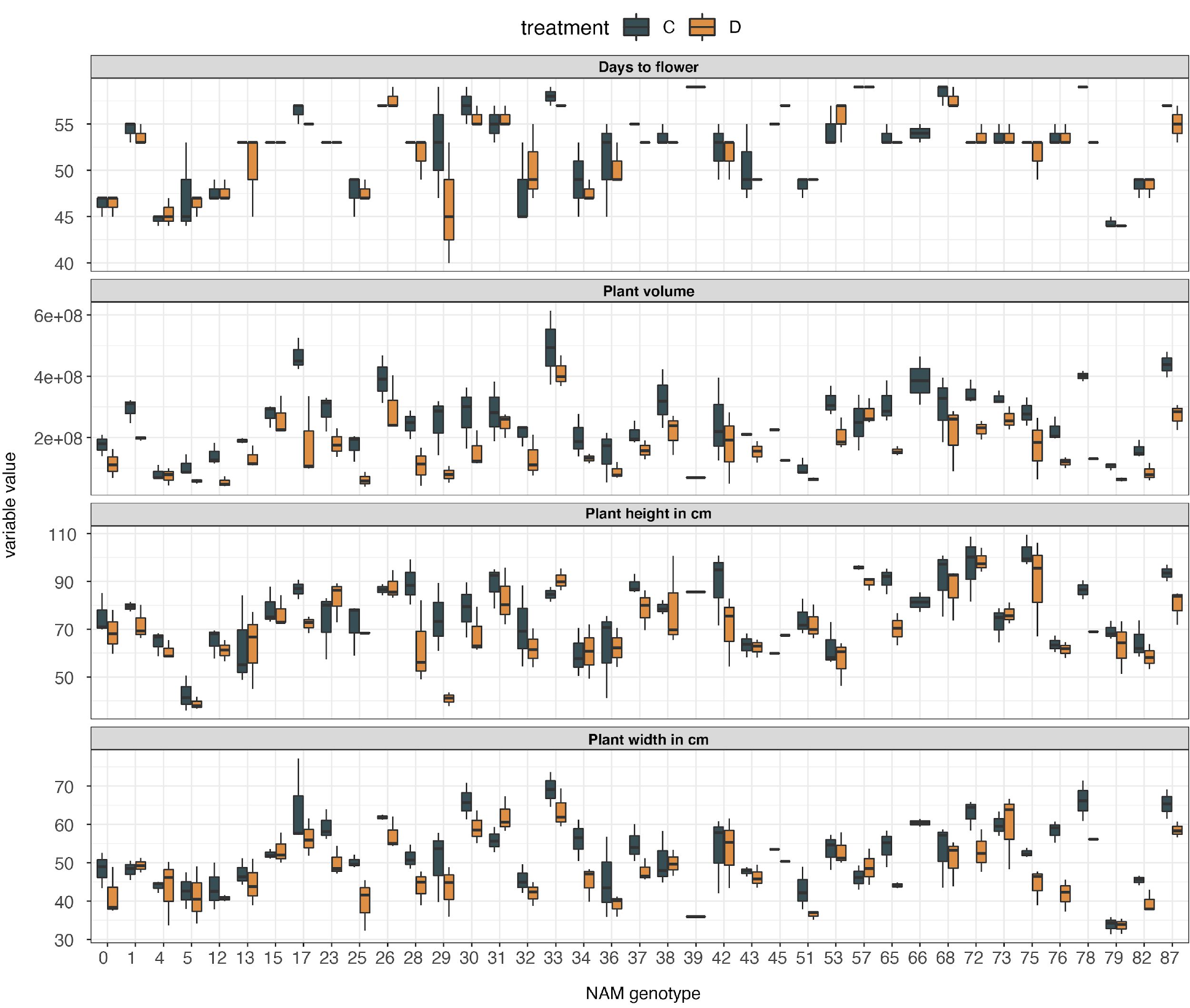


Figure S3.1: Variation among NAM genotypes and treatment groups of four exemplary growth traits. Plant volume is given in cubic pixels.


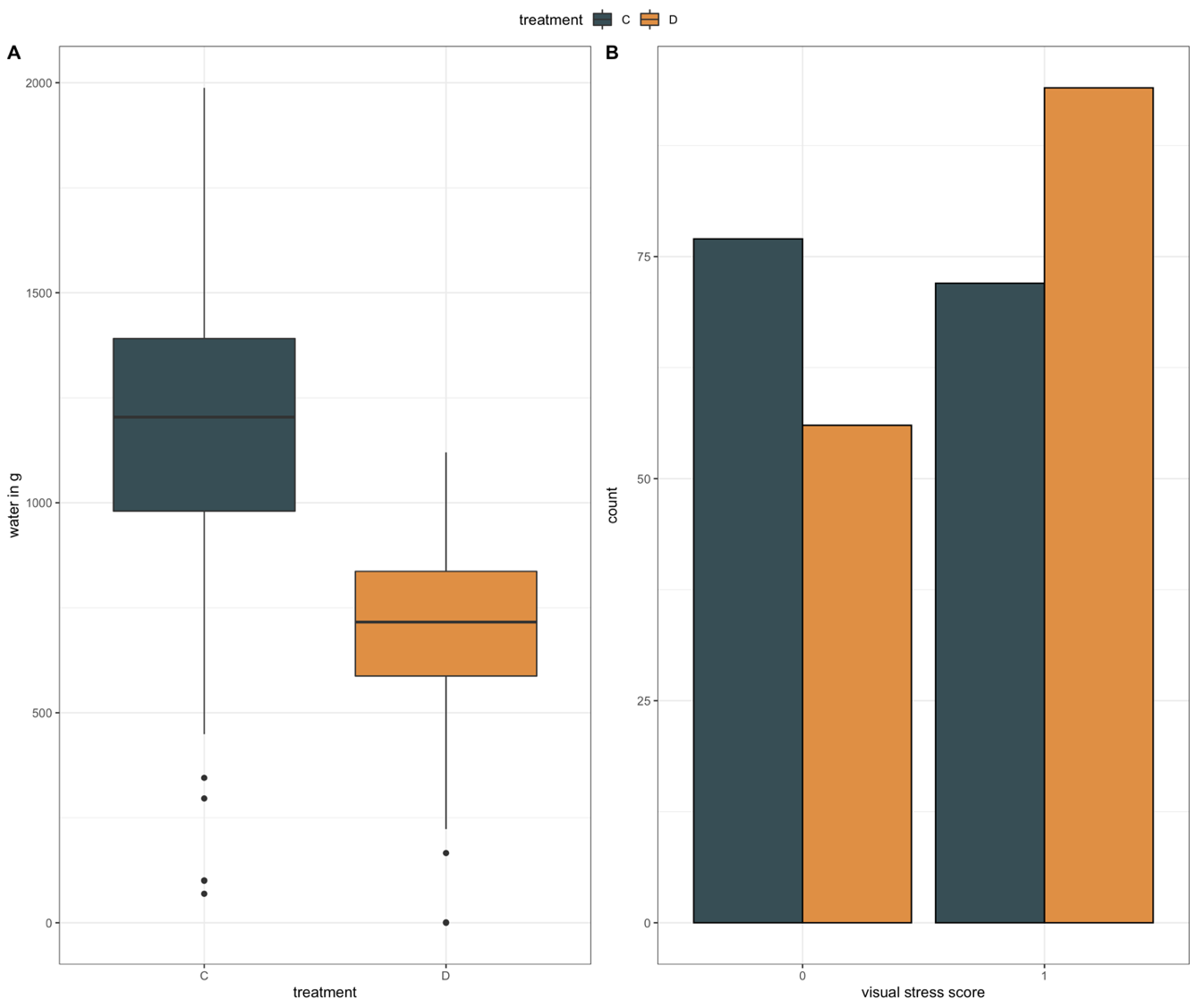


Figure S3.2. Administered water amounts (A) and visual stress (B) in well-watered (control) and water-limited (drought-treatment) groups on DAS 49 of *B.napus* LemnaTec phenotyping experiment. Control plants did receive higher amounts of water but with high variation, including several poorly watered outliers. Plants of each group displayed symptoms of drought stress.

*
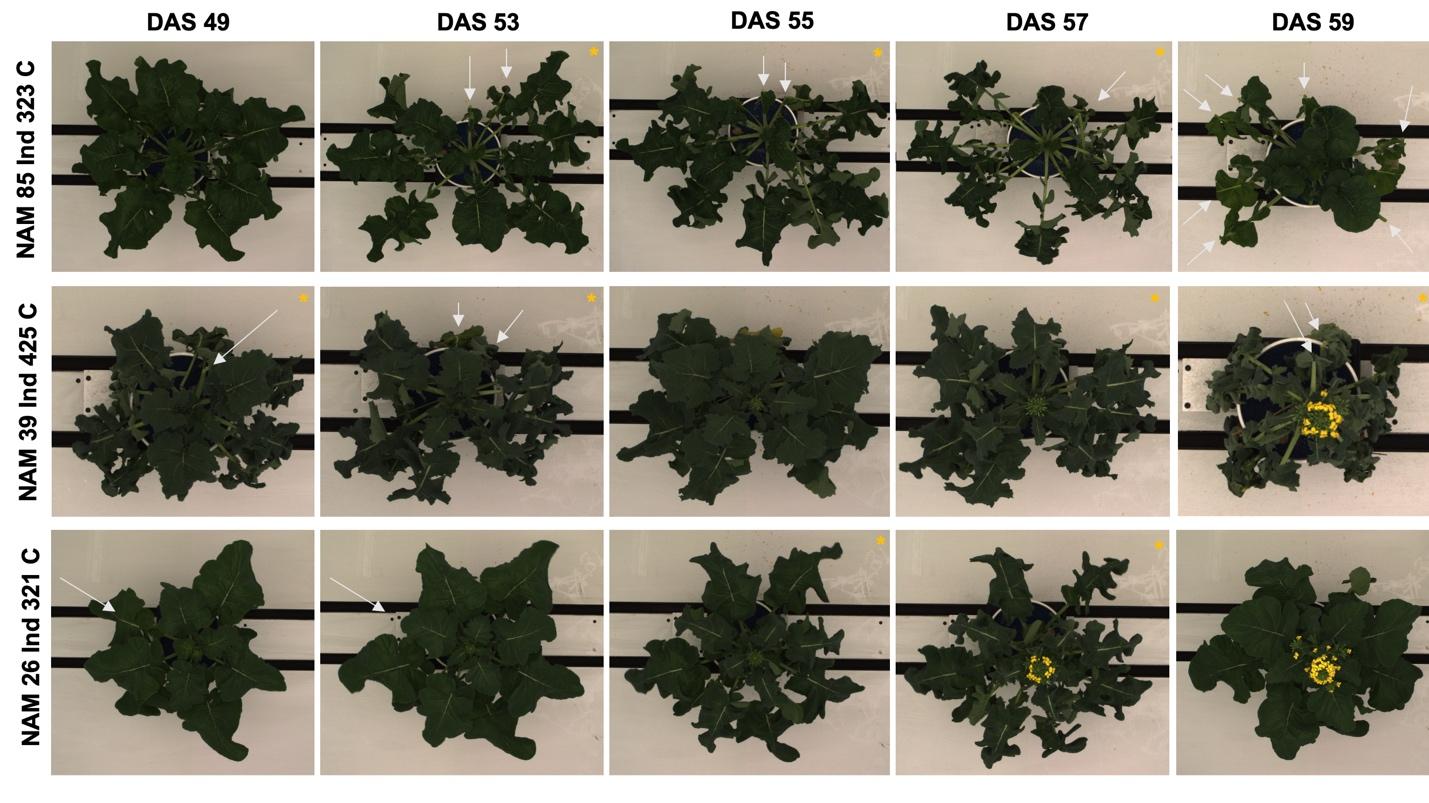
*

Figure S3.3: Examples of plants from the control group exhibiting drought stress symptoms (indicated by orange asterisk) and damage to larger rosette leaves (white arrows). See results and discussion in main text.


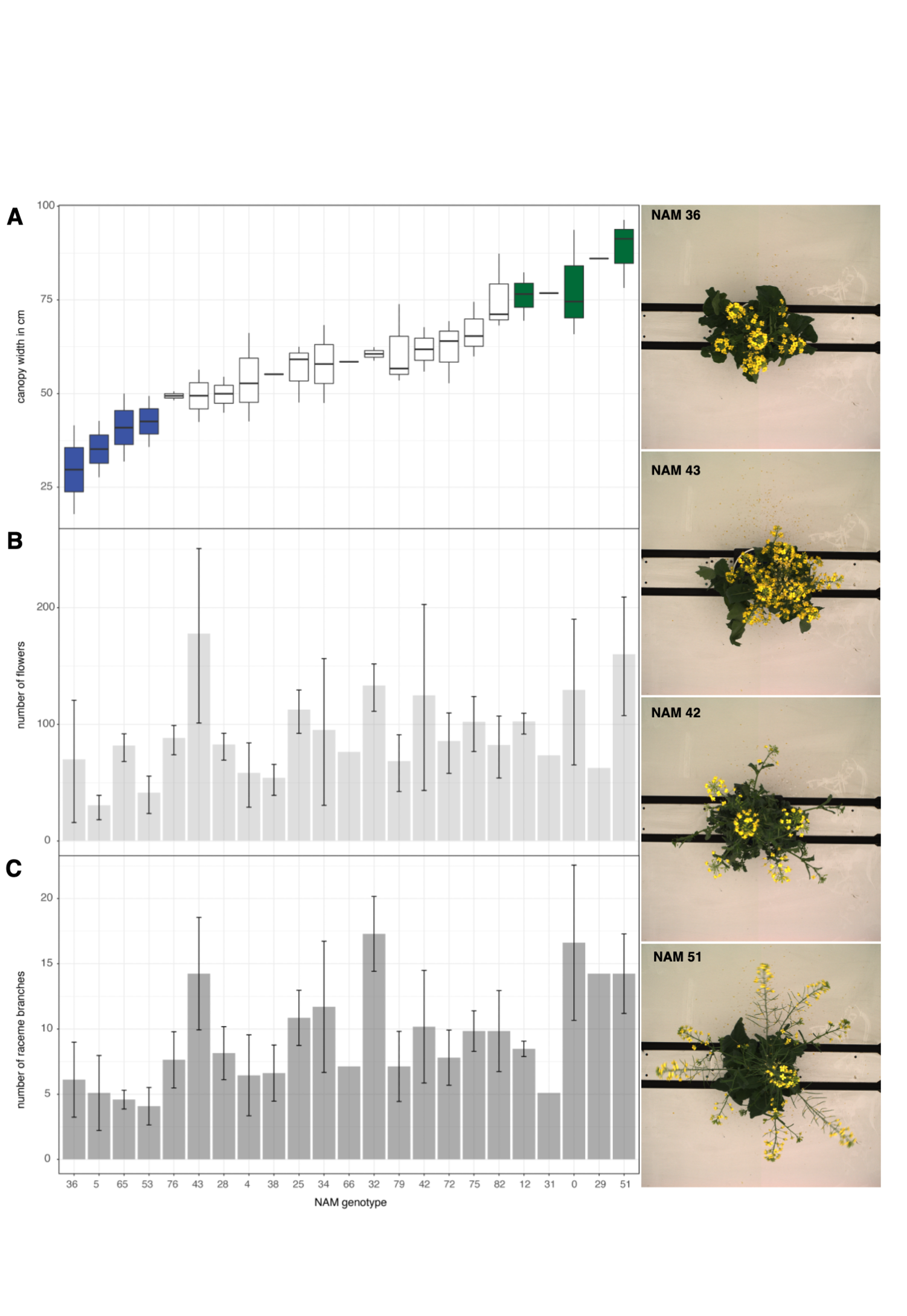


Figure S3.4: Comparison of canopy architecture of control plants on 4^th^ phenotyped day of flowering. Plot only includes genotypes that exhibited this stage of flowering. (A) Boxplots summarize maximum canopy width in cm. Dark blue boxes indicate genotypes with very compact inflorescences (mean plant width at least 10 cm greater than mean canopy width). Green boxes indicate genotypes with very loose inflorescences (mean canopy width at least 20 cm greater than mean plant width). White boxes are used for intermediate genotypes. (B) Bars show the mean number of flowers per genotype at this stage of flowering (whiskers give standard deviation). (C) Bars represent the mean number of raceme branches per genotype at this stage of flowering (whiskers give standard deviation). Images in the right panel show representative individuals exhibiting different canopy architecture.


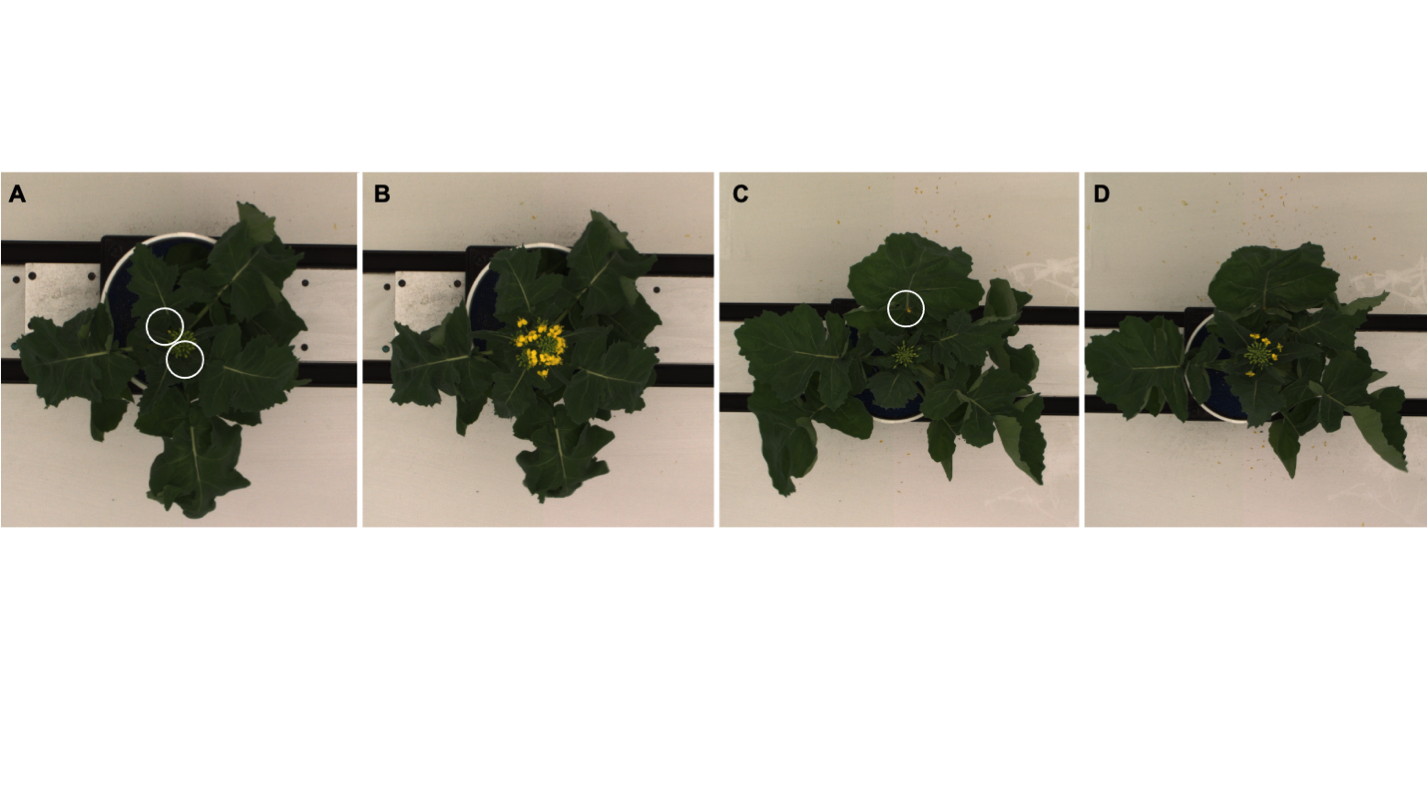


Figure S3.5: Examples of plants for which manual flower scoring and automatic detection were incongruent. (A) Flowers were automatically detected for this individual (NAM 43, Ind. 52, DAS 47), but manual scoring only recorded flowering on the next phenotyping time point (DAS 49), shown in (B). Fallen petals led to misdetection of flowering in (C, NAM 43, Ind. 539, DAS 53), while manual scoring recorded flowering only for the consecutive phenotyping time point (DAS 55), shown in (D).


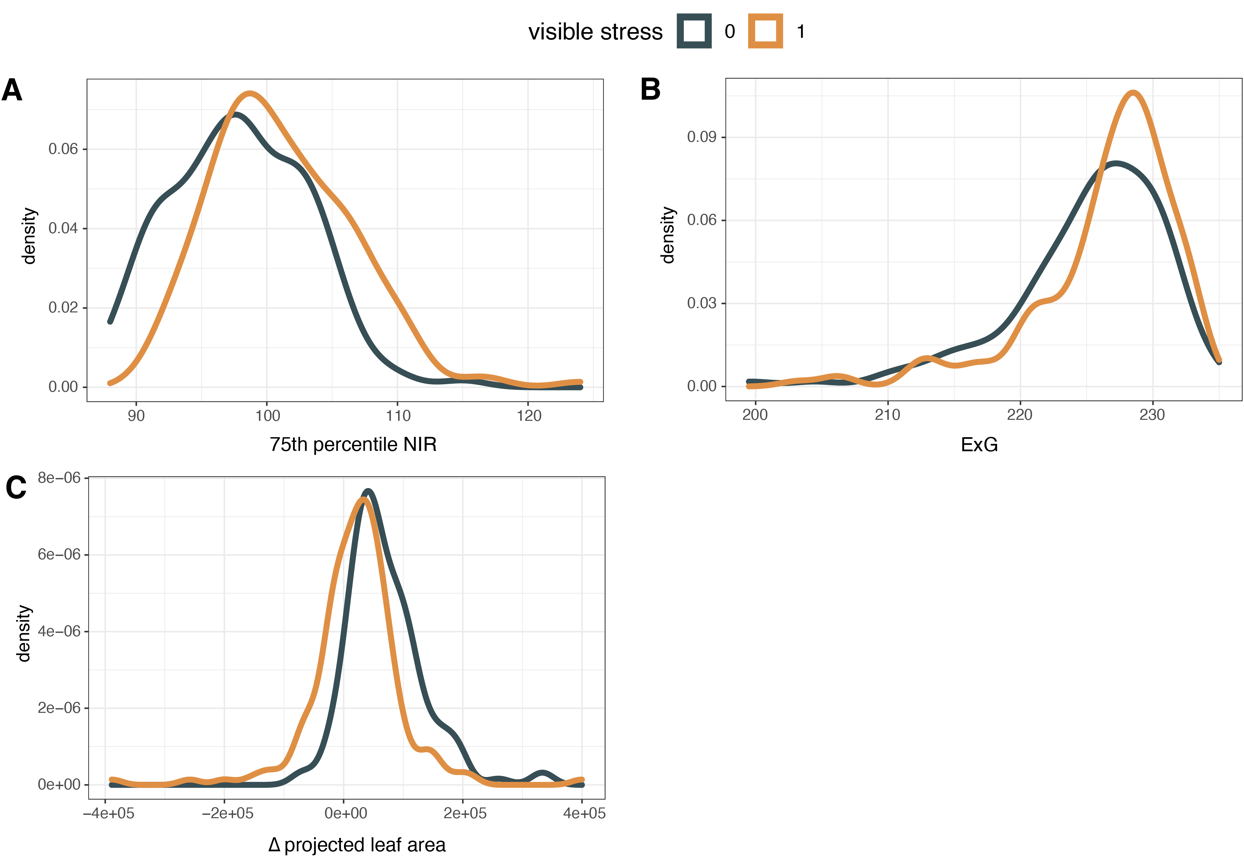


Figure S3.6: Density distributions of three tested parameters for differentiating between visibly drought stressed and non-stressed *B. napus* plants. Drought stress was scored manually on DAS 49 for all individuals (0: no visible stress, black line; 1: visible drought stress, orange line). (A) 75^th^ percentile NIR plant pixel intensity. (B) Excess green index (ExG) of plant area. (C) Change in projected leaf area (∆ pixels) from DAS 47 to DAS 49.


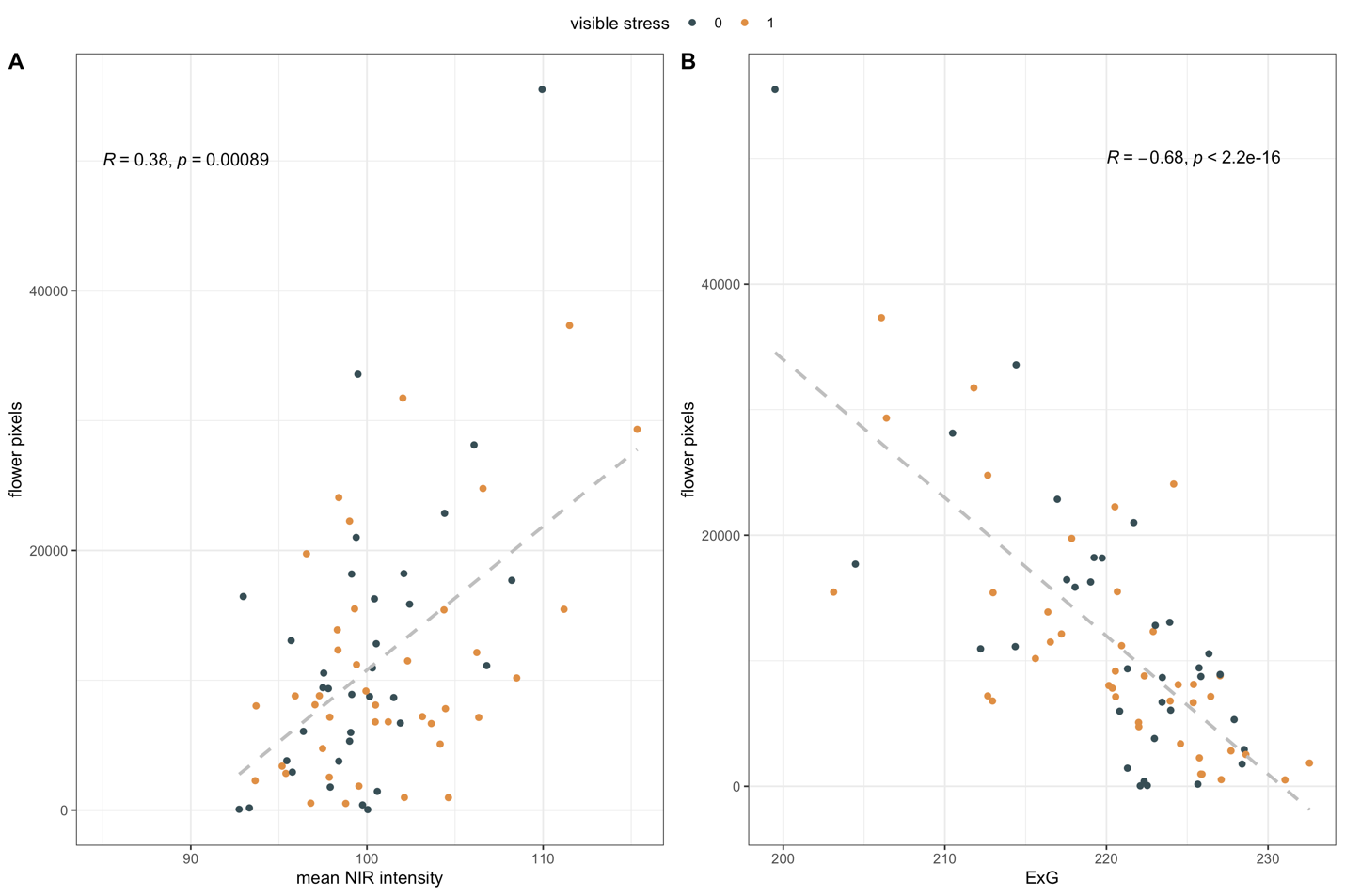


Figure S3.7: Relationships between the number of flower pixels and mean NIR intensity (A) as well as excess green index (B) on DAS 49. Only flowering individuals were included. Data points are coloured with respect to visual stress scores. Spearman's rank correlation rho and respective p-values are given at the top of each plot. Dashed lines show linear regressions.


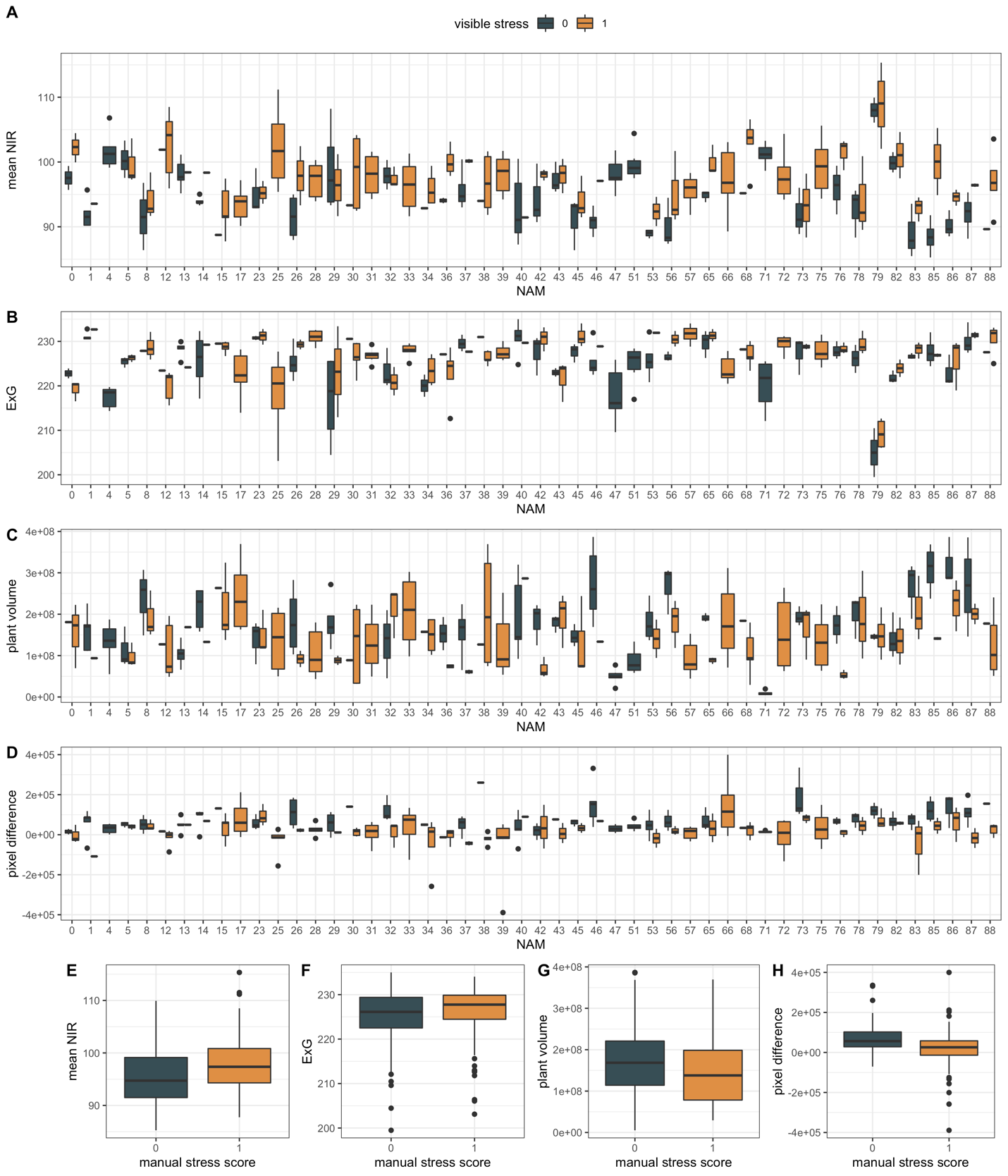


Figure S3.8: Genotypic differences in mean NIR intensity (A), excess green index (B), plant volume (C), and difference in plant pixel from one day to the next (D) on DAS 49. Boxes are coloured according to manually scored visible stress. Overall differences between visibly stressed and not stressed plants for mean NIR intensity (E), excess green index (F), plant volume (G) and pixel difference (H) are given below.
